# Ruthenium Drug BOLD-100 Regulates *BRAF*MT Colorectal Cancer Cell Apoptosis through AhR/ROS/ATR Signaling Axis Modulation

**DOI:** 10.1158/1541-7786.MCR-24-0151

**Published:** 2024-07-31

**Authors:** Daryl Griffin, Robbie Carson, Debbie Moss, Tamas Sessler, Deborah Lavin, Vijay K. Tiwari, Shivaali Karelia, Richard Kennedy, Kienan I. Savage, Simon McDade, Adam Carie, Jim Pankovich, Mark Bazett, Sandra Van Schaeybroeck

**Affiliations:** 1 Patrick G. Johnston Centre for Cancer Research, School of Medicine, Dentistry, and Biomedical Science, Queen’s University Belfast, Belfast, United Kingdom.; 2 Institute for Molecular Medicine, University of Southern Denmark, Odense, Denmark.; 3 Danish Institute for Advanced Study (DIAS), Odense, Denmark.; 4 Department of Clinical Genetics, Odense University Hospital, Odense, Denmark.; 5 Wellcome-Wolfson Institute for Experimental Medicine, School of Medicine, Dentistry, and Biomedical Science, Queens University Belfast, Belfast, United Kingdom.; 6 Bold Therapeutics Inc., Vancouver, British Columbia.

## Abstract

Patients with class I ^V600E^*BRAF*-mutant (MT) colorectal cancer exhibit a poor prognosis, and their response to combined anti-BRAF/EGFR inhibition remains limited. An unmet need exits for further understanding the biology of ^V600E^*BRAF*MT colorectal cancer. We used differential gene expression of *BRAF*WT and MT colorectal cancer cells to identify pathways underpinning *BRAF*MT colorectal cancer. We tested a panel of molecularly/genetically subtyped colorectal cancer cells for their sensitivity to the unfolded protein response (UPR) activator BOLD-100. To identify novel combination strategies for BOLD-100, we performed RNA sequencing and high-throughput drug screening. Pathway enrichment analysis identified significant enrichment of the UPR and DNA repair pathways in *BRAF*MT colorectal cancer. We found that oncogenic *BRAF* plays a crucial role in mediating the response to BOLD-100. Using a systems biology approach, we identified ^V600E^*BRAF*MT-dependent activation of the replication stress response kinase ataxia telangiectasia and Rad3-related (ATR) as a key mediator of resistance to BOLD-100. Further analysis identified acute increases in *BRAF*MT-dependent-reactive oxygen species levels following treatment with BOLD-100, which promoted ATR/CHK1 activation and apoptosis. Furthermore, activation of reactive oxygen species/ATR/CHK1 following BOLD-100 was mediated through the AhR transcription factor and CYP1A1. Importantly, pharmacological blockade of this resistance pathway with ATR inhibitors synergistically increased BOLD-100-induced apoptosis and growth inhibition in *BRAF*MT models. These results highlight a possible novel therapeutic opportunity for *BRAF*MT colorectal cancer.

**Implications:**

BOLD-100 induces *BRAF*MT-dependent replication stress, and targeted strategies against replication stress (e.g., by using ATR inhibitors) in combination with BOLD-100 may serve as a potential novel therapeutic strategy for clinically aggressive *BRAF*MT colorectal cancer.

## Graphical Abstract

**Figure:**
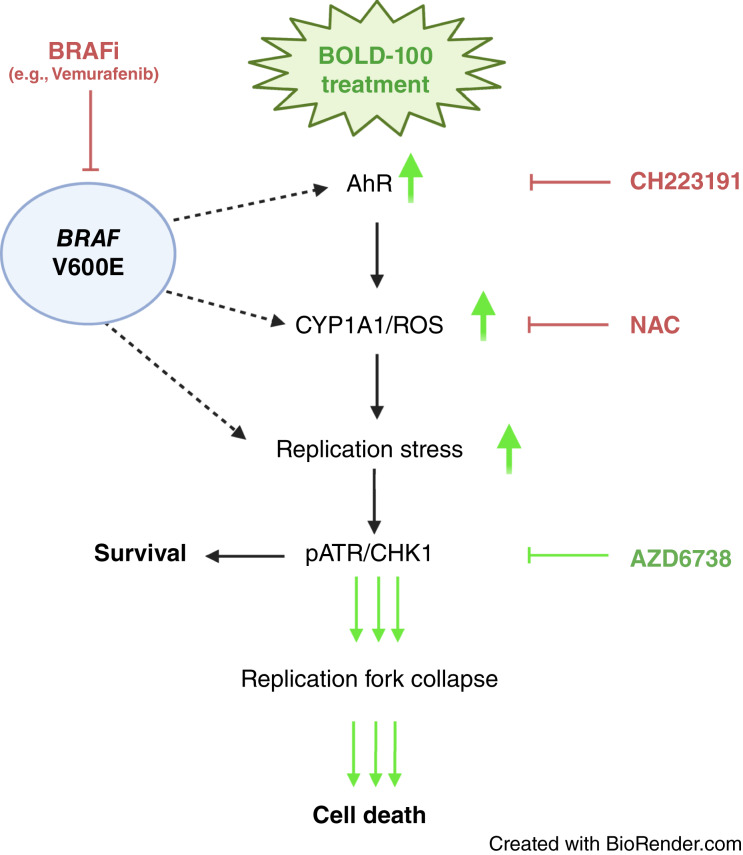

## Introduction

BRAF mutations are found in approximately 10% of metastatic colorectal cancer tumors, and around 90% of these tumors harbor a T1799A transversion in exon 15, resulting in a valine amino acid substitution (V600E; ref. 1). The ^V600E^*BRAF* (class I) mutation identifies a distinct subgroup of colorectal cancer, often associated with right sided tumors, microsatellite instability, spread to lymph nodes and peritoneum, and poor prognosis (2). Patients with ^V600E^*BRAF*MT colorectal cancer exhibit lower responses to chemotherapy. Despite meaningful clinical activity demonstrated in clinical trials with the novel BRAF/EGFR combination (3), not all patients will respond, and responses are often relatively short (4). Hence, ^V600E^*BRAF*MT colorectal cancer may also confer a potential target for new therapeutic approaches.

Molecular profiling of colorectal cancer datasets revealed endoplasmic reticulum (ER) stress related subtypes with adverse prognostic outcomes (5). The ER is a major organelle responsible for protein synthesis/folding (6). Protein misfolding results in ER stress and activation of the unfolded protein response (UPR), mediated by three key sensors, inositol-requiring protein-1 (IRE1), protein kinase RNA-like ER kinase (PERK), and activating transcription factor-6 (ATF6). Under basal conditions, the activation of these sensors is inhibited by binding of the ER-chaperone GRP78 to these three proteins (6). BOLD-100 [sodium trans-(tetrachlorobis (1H indazole) ruthenate (III))] is a first-in-class clinical-stage ruthenium-based small molecule drug that modulates the UPR through selective inhibition of GRP78. A phase I trial with BOLD-100 shows promising results for patients with colorectal cancer (7).

Herein, we report BRAF as a biomarker for response to BOLD-100 in colorectal cancer. Additional mechanistic studies identified a druggable resistance mechanism to BOLD-100 mediated by AhR via ROS and acute activation of the replication stress response kinase ATR (ataxia telangiectasia and Rad3-related), driven by oncogenic *BRAF*. Also, we show that concomitant treatment with ATR inhibitors and BOLD-100 leads to marked increases in therapeutic efficacy in *BRAF*MT colorectal cancer. Taken together, our results indicate that combined BOLD-100 with ATR inhibition might represent a valuable targeting strategy for ^V600E^*BRAF*MT colorectal cancer tumors.

## Materials and Methods

### Materials

BOLD-100 (7) is sodium trans-[tetrachlorobis (1H indazole) ruthenate (III)] manufactured with cesium as an intermediate salt form. Predecessor molecules including IT-139, NKP-1339, and KP1339. BOLD-100 was obtained from Bold Therapeutics Inc., vemurafenib (8), CH-223191 (9), AZD6738 (10), tapinarof (11), and binimetinib (3) from Selleck Chemicals LLC, M4344 (VX-803; ref. 12) and M6620 (berzosertib/VX-970; ref. 10) from Merck Healthcare KGaA, N-acetylcysteine (NAC; ref. 13) from Sigma-Aldrich, and encorafenib (3) from MedChem Express LLC. See Supplementary methods for references of non-FDA approved drugs used. ON-TARGETplus siRNA targeting *DDIT3*, siRNA’s targeting *ATR, CASP8, CASP9*, and the siRNA library were obtained from Invitrogen (Thermo Fisher Scientific) and Qiagen, respectively. See Supplementary methods for details of plasmid.

### Cell culture

All cell lines were screened monthly for Mycoplasma (MycoAlert Detection Kit, Lonza). Frozen stocks were immediately established from early passage cells. Cells were cultured for less than 20 passages following thawing. Authentication and culture of HT-29, VACO432/VT1, HCT116, RKO, SW620, GP5d, LoVo, LIM1215, LIM2405, and COLO205 cells have previously been described (14, 15). KM12 cells were obtained from the NCI-Frederick Cancer DCT Tumor repository (authentication: SNP arrays, oligonucleotide-base HLA typing, karyotyping, and STR). RW7213 cells were provided by Dr. Arango (University Hospital Vall d’Hebron; ref. 16). VACO432, VT1, and RKO were provided by Prof. Vogelstein (Johns Hopkins University School of Medicine). HDC8, C106, C125PM, SNU1411, OUSM23, HCC2998, WiDr, LIM2099, CX1, and OXCO3 colorectal cancer cells were obtained from Prof. Bardelli (17). HCT116 Caspase-8 wild-type and null paired cells were a gift from Prof. G. Lahav (Harvard Medical School; ref. 18). HCT116, HT-29, LS174T, DLD-1, LS513, LoVo, CCD-18, and COLO205 were purchased as authenticated stocks from ATCC (Authentication by short tandem repeat profiling/karyotyping/isoenzyme analysis). See Supplementary Methods for detailed protocols.

### Cell viability assays

Cell viability was determined using CellTiter-Glo (CTG, Promega) and Thiazolyl blue tetrazolium bromide (MTT) assays (Merck Life Sciences UK Limited; ref. 15), according to the manufacturer’s instructions.

### Flow cytometric analysis and cell death measurement

Apoptosis was evaluated using propidium iodide (PI, Sigma) staining to determine the percentage of cells with DNA content <2N (15). For annexin-V/PI analysis, cells were assessed according to the manufacturer’s instructions (BD Biosciences). PI, FITC-tagged annexin-V, and Hoechst stain (Invitrogen) were also used to quantify cell death through high content microscopy on an Array Scan XTI microscope (Thermo Fisher Scientific), as described previously (19).

### Western blotting

Western blotting has been described previously (15, 19). Details of antibodies are provided in Supplementary Table S1. Antibodies were used in conjunction with a HRP-conjugated antirabbit or antimouse secondary antibody. β-actin was used as loading control.

### Caspase-3/7-activity assays

Caspase-Glo3/7 activity assays (Promega) has been described previously (19).

### Total ROS detection

ROS production in cells was measured using the total ROS Assay Kit 520 nm (Thermo Fisher Scientific) according to the manufacturer’s instructions. Briefly, cells were seeded into 12 well-plates and incubated overnight. Cells were stained with 1× ROS assay stain and incubated for 1 hour after which cells were treated. At the experimental endpoint, cells were trypsinized and collected in PBS for analysis. To analyze geometric means for ROS staining, the BD FACS Calibur Flow Cytometer (BD Biosciences) and FlowJo v10.8.1 software were used. Total ROS levels were also assessed using the ROS-Glo H2O2 Assay Kit (Promega), according to the manufacturer’s instructions. Media in 96-wells was replaced with 80 µL of fresh medium, and 20 µL H_2_O_2_ substrate was added to each well and incubated at 37°C for 3 to 6 hours. Subsequently, 100 μL of ROS-Glo detection solution was added to each well and incubated for 20 minutes at room temperature in the dark. Luminescence signal was measured using the BioTek Synergy four microplate reader and normalized to cell number.

### siRNA and DNA transfections

siRNA and DNA transfections were carried out using HiPerfect (Qiagen) and X-tremeGENE (Merck), respectively, as described previously (15). See Supplementary methods for detailed protocol siRNA screen (Supplementary Table S2).

### RNA extraction and real-time reverse transcription-PCR analysis

RNA extractions were performed using the GeneJET RNA Purification Kit (Thermo Fisher Scientific). A260/280 and A260/230 ratios were utilized for quality control. RT-PCR was performed as previously described (19). Probes were purchased from Thermo Fisher Scientific. See Supplementary Methods for probe sequences.

### Data analysis

The data used in this study was obtained from the 155 Affymetrix U133 Plus 2.0 colorectal cancer cell line transcriptional profiles accessed through the NCBI GEO accession number GSE59857 and *BRAF* mutational status previously determined (20). Single sample gene set enrichment analysis (ssGSEA) on the 155 cell line microarray data was implemented through GenePattern web-tool (21) using the Hallmark gene set molecular signature (22). For heatmap visualization, a robust *Z*-score was calculated from the ssGSEA values. Mean *Z*-scores for the group of *BRAF*MT and *BRAF/KRAS*WT colorectal cancer cell lines for each hallmark pathway were presented in a heatmap using GraphPad Prism 10.1.

### RNA-sequencing

RNA sequencing (RNA-seq) of BOLD-100-treated VACO432 and VT1 cells was performed on the Illumina NextSeq500 sequencing platform, as described previously (15). Additional information is provided in the Supplementary methods.

### *In vivo* study

*In vivo* experiments were performed at Axis Bioservices, Northern Ireland, and were approved by the Axis Bioservices Animal Welfare and Ethical Review Committee, and all procedures were carried out under the guidelines of the Animal (Scientific Procedures) Act 1986. Details of the initial tolerability study are provided in the Supplementary Methods (Supplementary Fig. S4D). For the efficacy study, 5 × 10^6^ VACO432 cells were injected into the flank of male Balb/c nude mice. Mice received vehicle [0.9% saline with 10 mmol/L citrate buffer IV + (10% DMSO + 90% hydroxypropyl-β-cyclodextrin (10% made in PBS)) PO], BOLD-100 (50 mg/kg IV), AZD6738 (50 mg/kg PO), or BOLD-100 (50 mg/kg IV) with AZD6738 (50 mg/kg PO). Each treatment group contained eight animals. BOLD-100 was administered weekly and AZD6738 daily.

### Statistical analysis

Robust *Z*-scores (rZ = median/median absolute deviation) were calculated from cell viability assays. All data were plotted (mean and standard deviation, unless specified otherwise), and analyzed using GraphPad Prism 10.1. Significance was defined as *P* < 0.05; ^∗^, *P* < 0.01; ^∗∗^, *P* < 0.001; ^∗∗∗^, *P* < 0.0001; ^∗∗∗∗^; with *P* > 0.05 not significant (ns). Unless indicated otherwise, experiments are representative of three independent repeats. The nature of interaction between BOLD-100 and a second drug was determined by calculating combination index (CI) values according to the Chou–Talalay method (23), using CalcuSyn (Microsoft Windows). CI values <1, >1, and = 1 indicate synergy, antagonism, and additive effects, respectively.

### Data availability

Raw data for RNA-seq experiment have been deposited at the relevant NCBI platforms, under the accession number GSE252858.

## Results

### Oncogenic *BRAF* regulates sensitivity of colorectal cancer cells to BOLD-100

**Figure 1.**
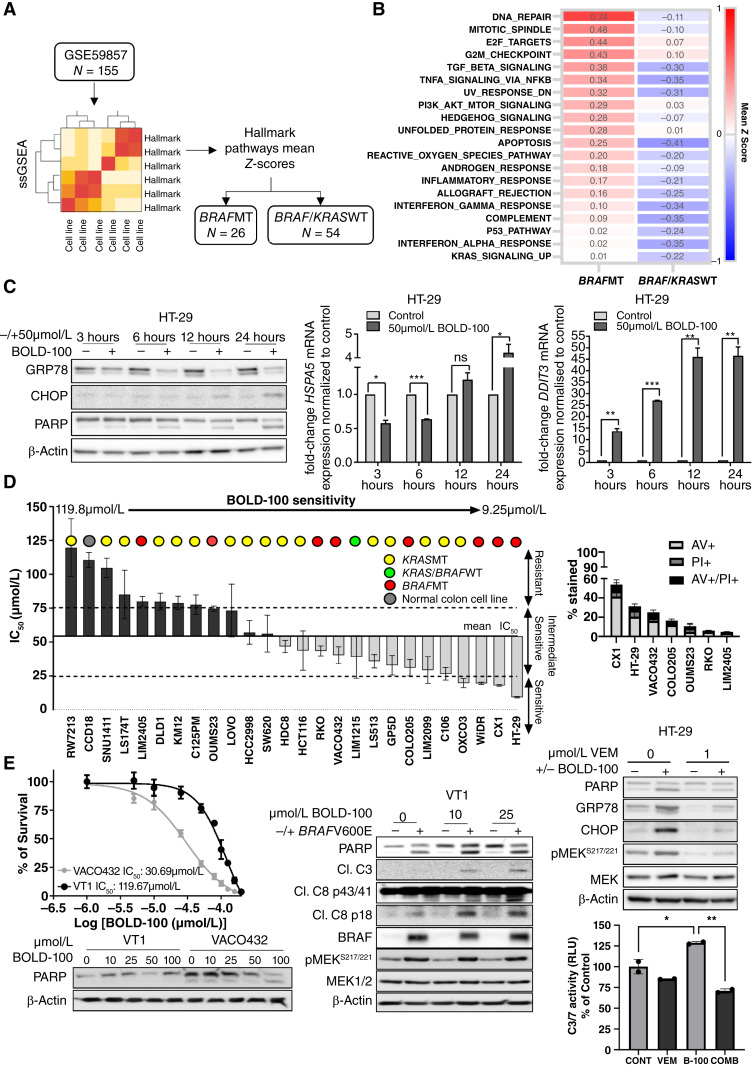
Sensitivity of colorectal cancer cell lines to BOLD-100. **A,** Consort diagram showing the 155 colorectal cancer GSE59857 cell line dataset selected for analysis of genes/pathways differentially expressed in *BRAF*MT and *KRAS/BRAF*WT colorectal cancer cell lines. **B,** GSE59857 heatmap visualizing the “cancer hallmarks pathways” (22) comparing *BRAF*MT and *BRAF/KRAS*WT colorectal cancer cells. Heatmap displays the mean *Z*-scores from single sample gene set enrichment analysis (red and blue rectangles). Top 20 pathways with largest mean *Z*-scores differences between BR*AF*MT and *BRAF/KRAS*WT colorectal cancer cells are presented, ranked according to the highest *Z*-score for *BRAF*MT colorectal cancer. **C,** HT-29 cells were treated with BOLD-100 for the indicated times. Left: GRP78, CHOP and PARP levels were determined by Western blotting (WB). *HSPA5* (middle) and *DDIT3* (right) mRNA were quantified using RT-PCR. Raw values were normalized to the expression of housekeeping genes *ACTB* and *GAPDH* and were analyzed using the ΔΔCT method. mRNA levels presented are relative to untreated control. **D,** Colorectal cancer cells (left) were treated with increasing concentrations of BOLD-100 for 72 hours and cell viability determined using CTG assay. IC_50_ values were calculated using Prism software package. Mean IC_50_ value of three independent experiments is shown. Dashed lines indicates 75% and 25% cell viability. *BRAF*MT colorectal cancer cells (right) were treated with 100umol/l BOLD-100 for 48 hours. Apoptosis was assessed using annexin-V/PI staining by high content screening. The graph indicates the percentage of positive stained cells. **E,***BRAF*MT VACO432 colorectal cancer cells, and its isogenic VT1 clone, were treated with increasing concentrations of BOLD-100. Cell viability was determined using CTG assays (top left) and PARP levels determined by WB (bottom left). WB analyses (middle) for PARP, cleaved caspase-3, caspase-8, BRAF, pMEK1/2^S217/221^ and MEK1/2 in VT1 cells transiently transfected with 1 µg of BRAFV600E expression construct for 12 hours followed by 48 hours treatment with BOLD-100. HT-29 cells (right) were preincubated with vemurafenib for 3 hours and thereafter treated with 50 μmol/L BOLD-100 for 48 hours and PARP, GRP78, CHOP, pMEK1/2^S217/221^, and MEK expression determined by WB (top). Apoptosis was assessed by caspase-3/7 activity assay (bottom). CONT, control; VEM, vemurafenib; B-100, BOLD-100.

To determine novel pathways associated with constitutively active ^V600E^*BRAF* and novel therapeutic strategies, we calculated hallmark pathway (22) mean *Z*-scores using the publicly available GSE59857 *BRAF*MT and *BRAF/KRAS*WT colorectal cancer dataset and the ssGSEA method (Fig. 1A). We selected the top 20 hallmark pathways altered between the two sample groups (Fig. 1B; Supplementary Table S3). Comparison of the pathway analyses results showed that DNA repair related pathways (DNA repair, mitotic spindle, E2F targets, G2M checkpoint, UV response DN, ROS pathway), UPR, apoptosis, TNFα signaling via NFκB, TGFβ, PI3K/mTOR, and Hedgehog signaling were markedly enriched in the *BRAF*MT colorectal cancer cell line group compared with the *BRAF/KRAS*WT colorectal cancer cells. Given our previous findings that combined ACY-1215/carfilzomib treatment result in acute ER stress and apoptosis in *BRAF*MT colorectal cancer (14) and the recent clinical development of the GRP78 inhibitor, BOLD-100, we carried forward the UPR pathway for further validation.

To test our hypothesis that *BRAF*MT colorectal cancer cells are vulnerable to specific modulation of the UPR, we analyzed the effect of BOLD-100 in a panel of 26 genetically characterized colorectal cancer cells. As expected, BOLD-100 effectively reduced GRP78 protein and mRNA expression and resulted in marked increases in *DDIT3* and *ATF4* mRNA levels (Fig. 1C; Supplementary Fig. S1A–S1C). Next, we quantified cell viability of each colorectal cancer cell line in our selected panel (Fig. 1D). We found that 8/26 (31%), 14/26 (54%), and 4/26 (15%) colorectal cancer cell lines showed resistance, moderate sensitivity, and sensitivity to BOLD-100, respectively. Among the most sensitive cells were the *BRAF*MT HT-29, CX1, and WiDr cells (IC_50_ 9.25–19.5 μmol/L), with *KRAS*^G12C^MT RW7213 and SNU1411 and the normal colonic myofibroblast cell line CCD-18Co (IC_50_ > 100 μmol/L) the most resistant cells. Among the *BRAF*MT colorectal cancer cells, only CX1, HT-29, and VACO432 cells showed marked induction of apoptosis after 48 hours of BOLD-100 treatment, as indicated by increases in annexin-V/PI staining [Fig. 1D (right); Supplementary Fig. S1D].

To investigate further the role of oncogenic *BRAF* in mediating response to BOLD-100, we evaluated the effect of BOLD-100 on viability of the parental *BRAF*MT VACO432 colorectal cancer cell line and its isogenic VT1 clone with a disrupted BRAF^V600E^ allele [Fig. 1E (left); ref. 24]. Sensitivity to BOLD-100 was markedly higher in the VACO432 cells (IC_50_: 30.69 μmol/L) compared with its WT clone (IC_50_: 119.67 μmol/L), and this was associated with increased apoptosis as determined by PARP cleavage in the VACO432 cell line compared with the VT1 cells. In addition, transient overexpression of *BRAF*V600E was associated with increased PARP and caspase-8/3 processing in response to BOLD-100 treatment in the *BRAF*WT VT1 cells [Fig. 1E (middle)]. Furthermore, treatment with the BRAF inhibitor vemurafenib abrogated BOLD-100-induced CHOP, GRP78, PARP cleavage, and caspase 3/7 activity [Fig. 1E (right)]. Collectively, these results would indicate that oncogenic *BRAF* regulates sensitivity to BOLD-100 treatment in colorectal cancer.

### A targeted TSG siRNA screen identifies loss of *caspase 8* as a mediator of BOLD-100 resistance

**Figure 2.**
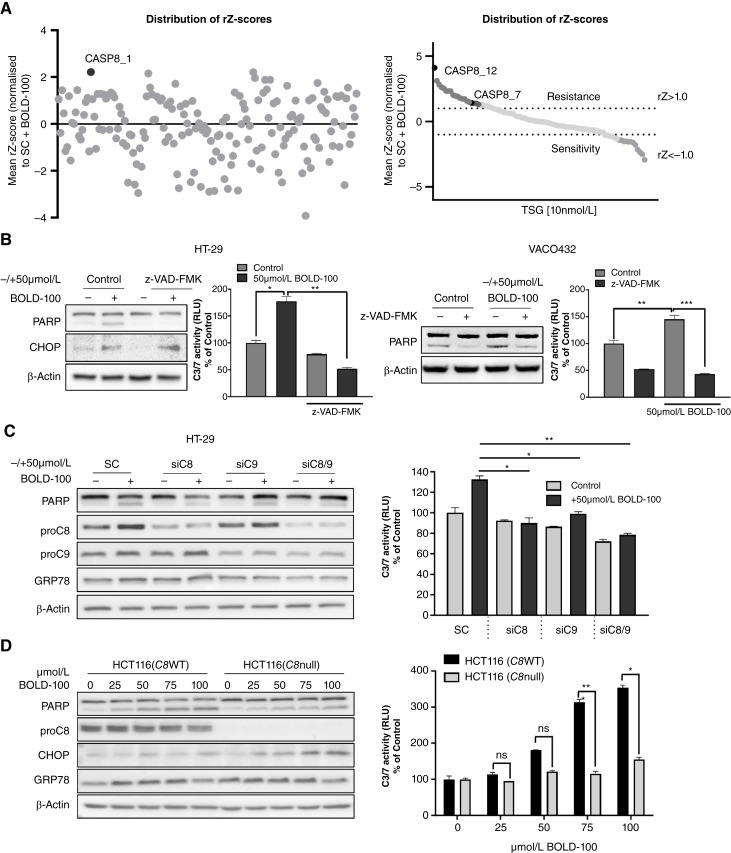
A targeted TSG siRNA screen identifies loss of *C8* as a mechanism of resistance to BOLD-100. **A,** Primary siRNA screen (left): HCT116 colorectal cancer cells were reverse transfected with siRNAs targeting 177 TSGs and 24 hours later treated with either DMSO control or 50 μmol/L BOLD-100. Cell viability was recorded 48 hours later using the CTG assay. Scatter plot showing distribution of *rZ*-scores for TSG siRNA screen. Positive scores indicate potential mediators of resistance to BOLD-100, whereas negative scores indicate potential mediators of sensitivity to BOLD-100. Secondary siRNA screen (right): HCT116 cells were reverse transfected with two additional independent siRNAs targeting 46 TSGs and positive hits from the primary screen. Waterfall plot showing distribution of *rZ*-scores. Each siRNA targeting *C8* is highlighted in black. **B,***BRAF*MT HT-29 (left) and VACO432 (right) cells were treated with BOLD-100 and preincubated with DMSO or the pan-caspase inhibitor (20 μmol/L). Apoptosis was determined by PARP and caspase 3/7 activity assay. **C,** HT-29 cells were transfected with 10 nmol/L *C8* or/and *C9* siRNA for 24 hours and thereafter treated with BOLD-100 for 24 hours. Apoptosis was assessed by WB analysis for PARP (left) and caspase-3/7 activity (right). Expression of pro-caspase-8, pro-caspase-9, GRP78 are also shown. **D,** Paired CRISPR HCT116*C8*WT and HCT116*C8*null cells were treated with increasing concentrations of BOLD-100 for 48 hours. Apoptosis was determined by WB for PARP (left) and caspase-3/7 activity levels (right). Expression of pro-caspase-8, GRP78, and CHOP are also shown.

To identify additional biomarkers for response to BOLD-100, we used a customized siRNA library targeting 177 tumor suppressor genes (TSG; Fig. 2A; Supplementary Fig. S2A; Supplementary Table S2). The effect of downregulating each of these TSGs on cell viability was tested in the easy-to-transfect *KRAS*MT HCT116 colorectal cancer cells (15), in the absence and presence of BOLD-100 treatment, and robust *Z*-score (rZ) values were calculated. Target genes were defined as resistance or sensitivity hits when siRNA demonstrated a *rZ*-score greater than 1 or less than −1 respectively. We identified 23 resistance and 23 sensitivity hits [Fig. 2A (left); Supplementary Fig. S2B] that were carried forward to a secondary siRNA screen incorporating an additional two independent siRNA sequences per target [Fig. 2A (right); Supplementary Fig. S2C]. This identified three positive resistance hits, and these were *Caspase-8* (mean *rZ*-score 2.6), *GLTSCR1* (mean *rZ*-score 1.95), and *RB1CC1* (mean *rZ*-score 1.7). To further validate the findings from the library screen, we knocked down *Caspase-8*, *GLTSCR1*, and *RB1CC1* in the *BRAF*MT HT-29 cells and investigated sensitivity to BOLD-100, using a cell viability assay (Supplementary Fig. S2D). Only silencing of *Caspase-8* resulted in significant decreased sensitivity to BOLD-100 compared with control siRNA. Additionally, caspase-dependent apoptosis following BOLD-100 was further assessed using the pan-caspase inhibitor z-VAD-FMK, which resulted in inhibition of BOLD-100-induced PARP and caspase-3/7 activity in HT-29 cells (Fig. 2B). To investigate further the relative importance of the extrinsic and intrinsic apoptotic pathways in mediating BOLD-100-induced apoptosis, HT-29 cells were transfected with si*Caspase-8* or si*Caspase-9* and treated with BOLD-100. Notably, si*Caspase-8* abrogated BOLD-100-induced PARP and caspase-3/7 activity (Fig. 2C). These results were confirmed using the caspase-8 null HCT116 cells (Fig. 2D). We previously reported that HT-29 cells behave in a type II manner (25). This model can explain why si*Caspase-9* also decreased BOLD-100-induced apoptosis in HT-29 cells. Interestingly, treatment with z-VAD-FMK did not affect BOLD-100-induced CHOP levels (Fig. 2B). Furthermore, silencing of CHOP did not decrease BOLD-100-induced PARP cleavage (Supplementary Fig. S2E), suggesting that the UPR pathway plays no role in the cell death following BOLD-100 treatment in *BRAF*MT colorectal cancer. Similar results were obtained in the *BRAF*MT VACO432 cell line [Fig. 2B (right); Supplementary Fig. S2F]. Collectively, these data suggest that the apoptosis induced by BOLD-100 proceeds via a caspase-8-mediated activation of the extrinsic apoptotic pathway.

### High-throughput drug screen reveals that pharmacological inhibition of ATR synergizes with BOLD-100 in *BRAF*MT colorectal cancer

**Figure 3.**
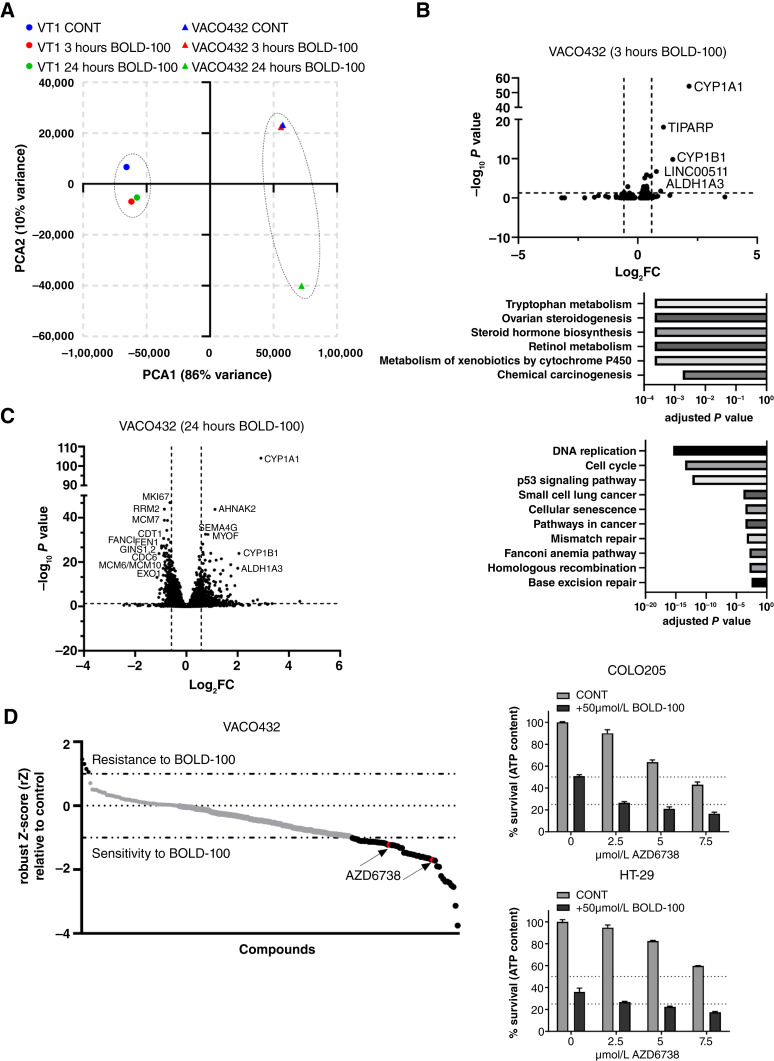
BOLD-100 rewires the signaling network of *BRAF*MT colorectal cancer cells. RNA-seq of BOLD-100 treated VACO432 and VT1 cells for 3 and 24 hours was performed. **A,** PCA plot displaying the separation between the *BRAF*MT VACO432 and VT1 *BRAF*WT clone following treatment with 24 μmol/L BOLD-100 for 3 and 24 hours. **B** and **C,** Volcano plots show the up- and downregulated genes following BOLD-100 treatment at the indicated time-points in VACO432 cells. Dashed lines on the *x* and *y*-axis indicate log_2_ratio of 0.58/−0.58, and −log_10_*P* value = 1.3, respectively. KEGGS pathway analysis was performed using DEG (FC: +/− 1.5 *P*: < 0.05) genelists for VACO432 cells treated for 3 and 24 hours with BOLD-100. **D,** VACO432 cells (left) were co-treated with 50 μmol/L BOLD-100 alone or combined with a panel of 61 small molecule inhibitors for 48 hours and cell viability assessed using the CTG assay. Three concentrations per drug were tested (Supplementary Table S5). Waterfall plot showing mean (*n* = 3) robust *Z*-scores (rZ) for each compound concentration used in the drug screen. Negative *rZ*-scores indicate agents that are sensitive to BOLD-100, and vice versa. Dashed lines on graphs indicate values of 1.0 and −1.0. *rZ*-scores for 2/3 concentrations for AZD6738 are highlighted in red. Positive hit from primary screen (right). *BRAF*MT COLO-205 and HT-29 colorectal cancer cells were treated with BOLD-100 alone or combined with AZD6738 for 72 hours and cell viability assessed using the CTG assay. Absolute cell viability is shown. Dashed line indicates 50% and 25% cell viability. Representative of three independent experiments are shown. CONT, control.

To gain further insight into the molecular mechanism-of-action and apoptosis following BOLD-100 treatment in *BRAF*MT colorectal cancer, we performed RNA-seq analysis in VACO432 and VT1 cells (Figs. 3A–C; Supplementary Figs. S3A–S3C; Supplementary Table S4; GSE252858). The gene expression data were merged into a single data set containing 35468 genes. Principal component analysis (PCA) clustering of these genes revealed that the greatest variability in gene expression was because of the *BRAF* status (Fig. 3A). A significant (*P* < 0.05) and potent [fold change (FC): >1.5] upregulation of the drug metabolizing cytochrome P450 genes (e.g., *CYP1A1*) was observed 3 and 24 hours post-BOLD-100 treatment in the VACO432 cells (Fig. 3B and C). Furthermore, significant downregulation of the E2F target gene MKI67, encoding the proliferation marker Ki-67, and genes involved in DNA biosynthesis and repair (e.g., *RRM2*, *FANCI*, *MCM7*, *FEN1*, and *EXO1*) was observed 24 hours following BOLD-100 treatment in the VACO432 cells (Fig. 3C). RT-PCR analysis confirmed marked decreases in *RRM2*, *FANCI*, and *FEN1* mRNA levels following BOLD-100 treatment (Supplementary Fig. S3D). To identify pathways that are involved in the molecular mechanisms-of-action and resistance to BOLD-100 treatment, KEGG pathway analysis was performed using the gene lists generated for the VACO432 cells (Supplementary Table S4). These results showed that tryptophan metabolism, steroid biosynthesis/ovarian steroidogenesis, retinol metabolism, and metabolism of xenobiotics by cytochrome P450 were the top five pathways identified from the 3 hours post-BOLD-100 gene list (Fig. 3B). Additionally, a significant enrichment of gene sets in DNA replication, cell cycle, and *TP53* signaling pathways was observed using the 24 hours post-BOLD-100 gene list (Fig. 3C). No significant changes in BOLD-100-induced gene expression (FC: >1.5 or < −1.5) were observed in the *BRAF*WT clone (Supplementary Fig. S3C).

Next, we performed a focused drug screen to identify compounds that could effectively suppress viability of *BRAF*MT colorectal cancer cells when combined with BOLD-100. We used a drug library targeting the top druggable KEGG pathways previously identified (Supplementary Table S4—24 hours BOLD-100; Supplementary Fig. S3E). On the basis of potential for clinical application, we prioritized 61 compounds (Supplementary Table S5). The effect of these drugs in the absence and presence of BOLD-100 was tested in the *BRAF*MT VACO432 cells. Next, positive hits were identified as compounds that resulted in robust *Z*-scores less than −1.0 in three independent experiments for ≥2/3 concentrations used; this identified 12 hits [Fig. 3D (left)] and included the ATR inhibitor AZD6738, Mre11–Rad50–Nbs1 (MRN) complex inhibitor Mirin, DNA-PK inhibitor NU7441, autophagy kinase ULK1 inhibitor SBI-0206965, inducers of apoptosis TRAIL, ABT737 and Entinostat, Wnt pathway activator CHIR-99021, MEK1/2 inhibitor AZD6244, EGFR/HER2 inhibitor lapatinib, and chemotherapeutics doxorubicin and vinorelbine tartrate. Of these, only AZD6738 resulted in potent decreases in absolute cell viability of > 75%, when combined with BOLD-100 across all the concentrations analyzed (Supplementary Fig. S3F). To exclude cell line-specific effects, we extended these studies to the *BRAF*MT HT-29 and COLO-205 cell line and also confirmed strong decreases in cell viability for the BOLD-100/AZD6738 combinations [Fig. 3D (right)]. Collectively, these data indicate that concomitant suppression of the ATR kinase survival axis is needed for a robust antisurvival response following BOLD-100 treatment in *BRAF*MT colorectal cancer.

### ATR inhibitors AZD6738, M4344 and M6620 potently synergizes with BOLD-100 in *BRAF*MT colorectal cancer

**Figure 4.**
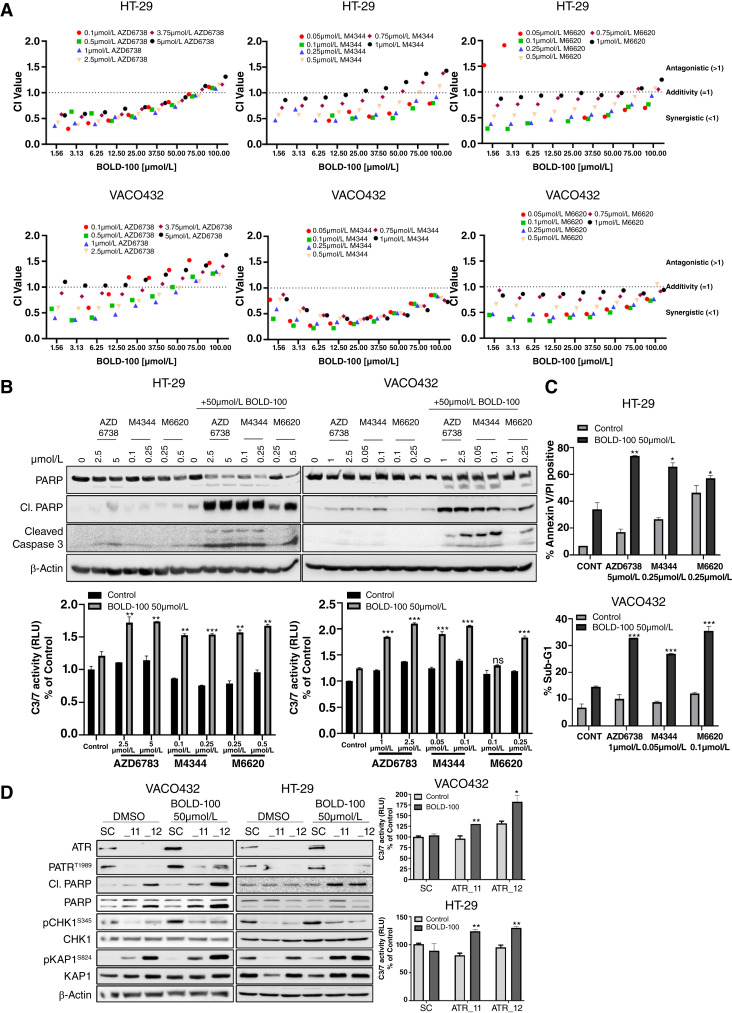
Combined ATR inhibition with BOLD-100 results in apoptosis in *BRAF*MT colorectal cancer. **A,** CTG cell viability assays in *BRAF*MT colorectal cancer cells co-treated with BOLD-100 and ATR inhibitors (AZD6738, M4344, M6620) for 72 hours. CI values were calculated to evaluate the nature of interaction. **B,** PARP, cleaved PARP and cleaved caspase-3 (top), and caspase-3/7 activity levels (bottom) in *BRAF*MT cells co-treated with BOLD-100 and ATR inhibitors for 48 hours. **C,** HT29 and VACO432 cells were co-treated with BOLD-100 and ATR inhibitors for 48 hours, and apoptosis was assessed using PI or annexin-V/PI staining. The graph indicates the percentage of positive stained cells. **D,** VACO432 and HT-29 cells were transfected with 10 nmol/L of SC or two different siRNA sequences targeting *ATR* for 24 hours, followed by treatment with BOLD-100 for 48 hours and apoptosis assessed by WB for PARP, Cleaved PARP (left) and Caspase 3/7 activity assay (right). Expression of pATR^T1989^, ATR, pCHK1^S345^, CHK1, pKAP1^S824^, and KAP was also determined.

We validated these drug screening results using synergy studies, using ATR inhibitors AZD6738, M4344, and M6620. Calculation of CI values confirmed moderate/strong synergy between AZD6738, M4344, or M6620 and BOLD-100 for the majority of combinations in *BRAF*MT HT-29 and VACO432 cell lines (Fig. 4A). Similar results were obtained in a wider panel of *BRAF*MT (COLO-205, RKO, LIM2405) cell lines (Supplementary Fig. S4A). Furthermore, combined AZD6738/BOLD-100 treatment markedly decreased the colony-forming ability of *BRAF*MT colorectal cancer cells (Supplementary Fig. S4B). Additionally, combination of pharmacological inhibition of ATR with BOLD-100 resulted in potent increases in apoptosis as indicated by increased PARP and caspase-3 cleavage in *BRAF*MT colorectal cancer cells [Fig. 4B (top)]. These results were confirmed quantitatively using caspase-3/7 activity assays and annexin-V/PI staining [Fig. 4B (bottom) and 4C]. Similar effects were observed in the *BRAF*MT COLO205, LIM2405, and RKO cells (Supplementary Fig. S4C). In addition, ATR targeted siRNA combined with BOLD-100 resulted in significant increases in apoptosis in *BRAF*MT colorectal cancer cells (Fig. 4D). Functional knockdown of ATR was confirmed by examining phosphorylation of CHK1-Ser345, a bona fide ATR target (Fig. 4D). Taken together, these results would suggest that combined ATR inhibition/BOLD-100 treatment could result in objective responses in a clinical setting in *BRAF*MT colorectal cancer (26).

Next, we assessed the *in vivo* therapeutic efficacy of combined AZD6738/BOLD-100 treatment (Supplementary Fig. S4D). We selected the VACO432 *BRAF*MT colorectal cancer model, which previously showed exponential growth characteristics when grown as xenografts (27). The VACO432 model was resistant to single agent BOLD-100 treatment and exhibited slowed but persistent growth when mice were treated with AZD6738 [Supplementary Fig. S4E (top)]. Although combination treatment of BOLD-100/AZD6738 resulted in further reduction in tumor growth, compared with single agent AZD6738, no tumor regression was observed. Treatment with AZD6738 and BOLD-100/AZD6738 was less well tolerated in this mouse model as shown by decreases in body weight over the 3-weeks of treatment [Supplementary Fig. S4E (bottom)]. This resulted in AZD6738 treatment interruptions of 2 to 3 days for five and six mice in AZD6738 and BOLD-100/AZD6738 groups, respectively.

### Effect of BOLD-100 on ATR/CHK1 activation in *BRAF*MT colorectal cancer

**Figure 5.**
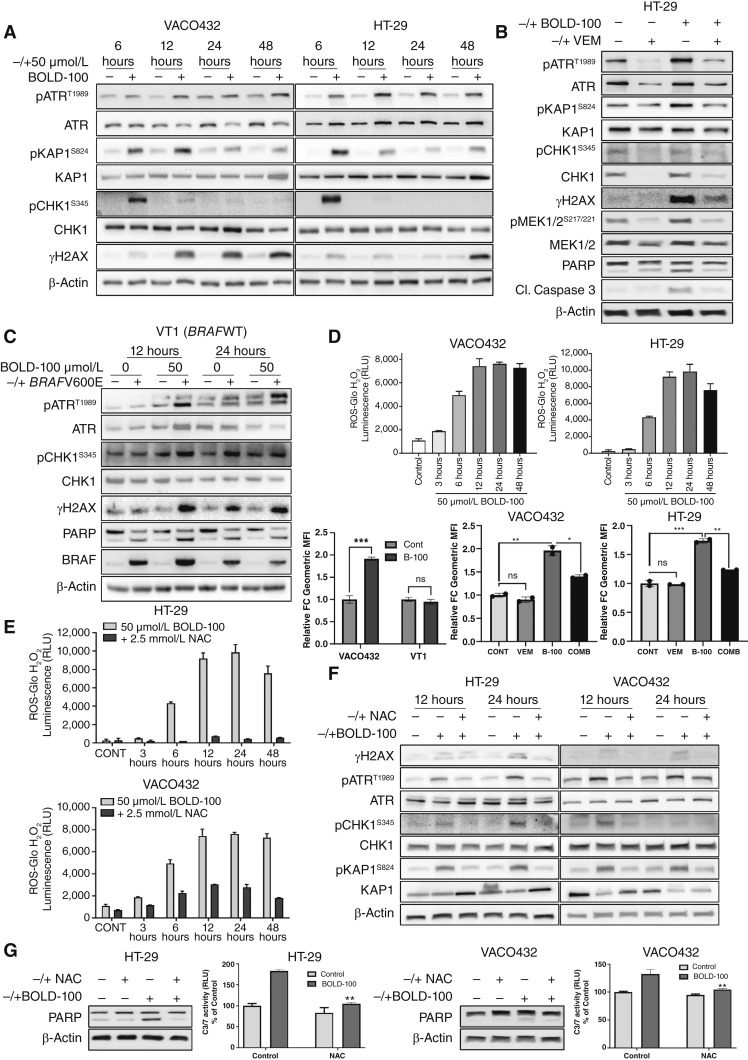
Oncogenic *BRAF* regulates BOLD-100 induced ROS, ATR/CHK1 activation and cell death. **A,** HT-29 and VACO432 cells were treated with BOLD-100 for the indicated times and pATR^T1989^, ATR, pKAP1^S824^, KAP1, pCKH1^S345^, CHK1, and γH2AX levels determined by WB. **B,** HT-29 cells were preincubated with 1umol/l vemurafenib for 3 hours and thereafter treated with 50 μmol/L BOLD-100 for 48 hours and pATR^T1989^, ATR, pKAP1^S824^, KAP1, pCKH1^S345^, CHK1, γH2AX, pMEK1/2^S217/221^, MEK, PARP, and cleaved caspase-3 expression determined by WB. **C,** WB analyzes for pATR^T1989^, ATR, pCKH1^S345^, CHK1, γH2AX, PARP, and BRAF in VT1 cells transiently transfected with 1 µg of BRAFV600E expression construct for 12 hours followed by treatment with BOLD-100 for the indicated times. **D,** VACO432 and HT-29 cells (top) were treated with BOLD-100 for indicated times and ROS levels assessed using the ROS-Glo H_2_O_2_ Assay Kit. ROS detection (bottom left) by flow cytometry in VACO432 and VT1 cells following treatment with 50 μmol/L BOLD-100 for 48 hours. ROS detection  (bottom middle/right) by flow cytometry in VACO432 and HT-29 cells, preincubated with vemurafenib for 3 hours, followed by 48 hours treatment with 50 μmol/L BOLD-100. MFI, mean fluorescence intensity. **E,** Cells were preincubated with NAC for 6 hours and thereafter treated with BOLD-100 for 48 hours and ROS levels assessed using the ROS-Glo H_2_O_2_ Assay Kit. **F,** Cells were preincubated with 5 mmol/L NAC for 6 hours and thereafter treated with BOLD-100 for the indicated times and γH2AX, pATR^T1989^, ATR, pKAP1^S824^, KAP1, pCKH1^S345^, and CHK1 levels determined by WB. **G,** Colorectal cancer cells were preincubated with 5 mmol/L NAC for 6 hours and thereafter treated with BOLD-100 for 48 hours and apoptosis was assessed by WB analysis for PARP (left) and caspase-3/7 activity assays (right). CONT, control; VEM, vemurafenib; B-100, BOLD-100.

To gain insight into the mechanisms involved in regulating the interaction between BOLD-100 and AZD6738, we examined how BOLD-100 affects the ATR/CHK1 replication stress response pathway using Western blotting (Fig. 5A). We found increased ATR Thr-1989 phosphorylation (an autophosphorylation site; ref. 28) as early as 6 hours following treatment with BOLD-100 in both *BRAF*MT colorectal cancer cells (Fig. 5A). Accordingly, we observed a marked increase in ATR mediated CHK1-Ser345 phosphorylation (29), 6 hours after BOLD-100 treatment (Fig. 5A). Also, we observed pKAP1-Ser824 indicating DNA double strand break induced ATM activation (KAP1-Ser824 is solely targeted by ATM; ref. 29) as early as 6 hours following BOLD-100. In line with this, phosphorylated H2AX (γH2AX) levels were acutely increased in response to BOLD-100 treatment (Fig. 5A). RPA32-Ser33 phosphorylation levels, the primary phosphorylation target of ATR in response to replication stress (30), was also upregulated in response to BOLD-100 treatment [Supplementary Fig. S5A (top left)]. Notably, co-treatment with AZD6738 and BOLD-100 abrogated BOLD-100-induced pATR/pCHK1 levels and resulted in marked upregulation in γH2AX levels [Supplementary Fig. S5A (bottom left and top right)]. Intriguingly, KAP1-Ser824 phosphorylation was upregulated in ATR depleted cells (Fig. 4D), which was further increased following BOLD-100 treatment. This would suggest that loss of ATR combined with BOLD-100 results in double stranded DNA breaks, triggering cell death. Importantly, BOLD-100-induced pATR/pCHK1/γH2AX levels were markedly higher in the *BRAF*MT VACO432 cell line, compared with the levels observed in its isogenic *BRAF*WT clone [Supplementary Fig. S5B (top left)]. Moreover, preincubation with vemurafenib potently decreased BOLD-100 induced pATR/γH2AX levels and abrogated BOLD-induced apoptosis in *BRAF*MT colorectal cancer cells [Fig. 5B; Supplementary Fig. S5B (top right)]. Similar results were obtained with the MEK1/2 inhibitor binimetinib [Supplementary Fig. S5B (middle left)]. In addition, transient overexpression of *BRAF*V600E led to increased basal and BOLD-100-induced pATR/γH2AX levels and was associated with increased PARP processing in response to BOLD-100 treatment in the *BRAF*WT VT1 cells (Fig. 5C).

Previous studies showed that metal-based anticancer chemotherapeutics, such as ruthenium compounds, can cause multi-modal lethal damage, including by inducing ROS-mediated DNA damage (31). Therefore, we evaluated the involvement of ROS in regulating BOLD-100-induced pATR/pCHK1/γH2AX levels and cell death in *BRAF*MT colorectal cancer. We found that BOLD-100 treatment resulted in a time-dependent and significant accumulation of intracellular ROS in *BRAF*MT VACO432 and HT-29 cells, but not in the isogenic VT1 clone (Fig. 5D). Importantly, treatment of *BRAF*MT colorectal cancer cells with vemurafenib significantly decreased BOLD-100-induced ROS levels [Fig. 5D (bottom)]. Similar results were obtained with binimetinib and the BRAF inhibitor encorafenib (Supplementary Fig. S5B). ROS production following BOLD-100 treatment in *BRAF*MT colorectal cancer cells was confirmed using the ROS scavenger NAC (Fig. 5E; ref. 13). Furthermore, exposure of *BRAF*MT cells to NAC abrogated the increased pATR/pCHK1/γH2AX levels and decreased cell viability following BOLD-100 treatment, and this resulted in marked reduction of BOLD-induced apoptosis, as indicated by reduction in PARP cleavage and caspase-3/7 activity (Fig. 5F and G; Supplementary Fig. S5C). Collectively, these results indicate that the ROS-mediated pATR/pCHK1 survival response following BOLD-100 treatment is driven by oncogenic *BRAF*.

### The AhR regulates BOLD-100-induced ATR/CHK1/γH2AX activation and survival in *BRAF*MT colorectal cancer cells

**Figure 6.**
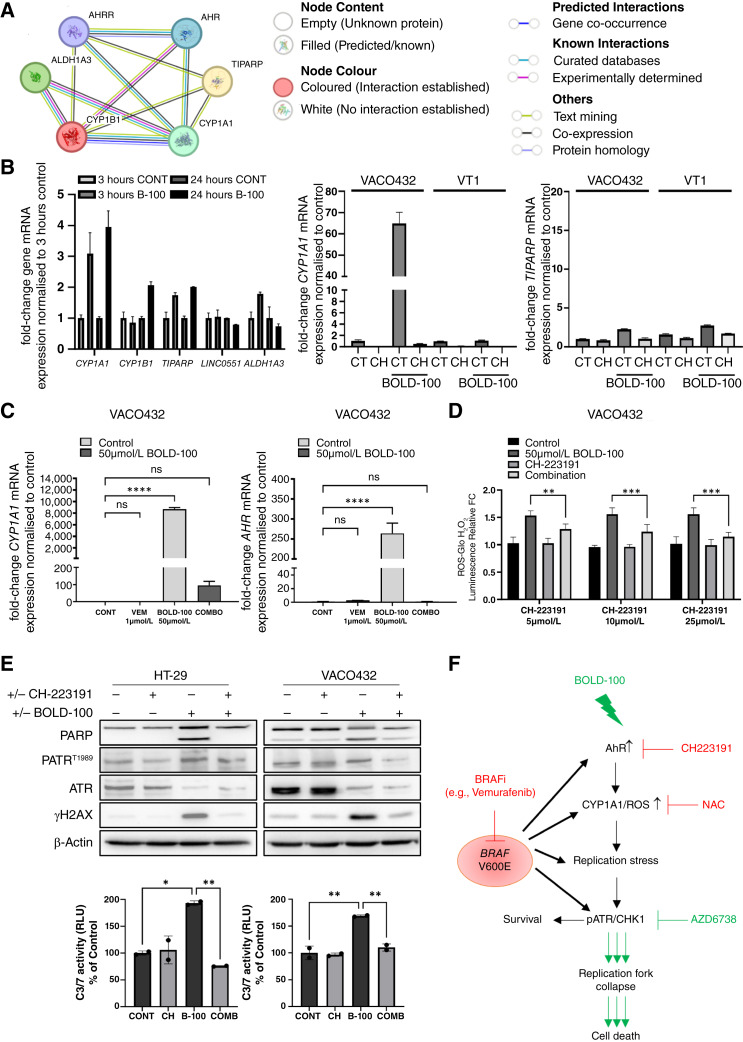
BOLD-100 regulates apoptosis through *BRAF*MT-dependent AhR/CYP1A1/ROS/ATR axis activation. **A,** String network formed by the significant upregulated genes, 3 hours following BOLD-100 treatment in VACO432 cell line, form a cluster around AhR. **B,** VACO432 cells (left) were treated for indicated times with 50 μmol/L BOLD-100. *CYP1A1, CYP1B1, TIPARP, LINC0051, *and* ALDH1A3* mRNA were quantified using RT-PCR. Raw values were normalized to the expression of housekeeping genes *ACTB* and *GAPDH* and were analyzed using the ΔΔCT method. mRNA levels presented are relative to SC. VACO432 and VT1 cells (right) were pretreated with 10 μmol/L CH-223191 (CH) for 3 hours and thereafter treated with 50 μmol/L BOLD-100 for 3 hours, and *CYP1A1* (center) and *TIPARP* (right) mRNA was quantified using RT-PCR. CT, control. **C,** VACO432 cells were preincubated with vemurafenib (VEM) for 3 hours followed by BOLD-100 treatment for 24 hours and *CYP1A1* and *AhR* mRNA was quantified using RT-PCR. **D,** VACO432 cells were pretreated with CH-223191 at indicated concentrations for 3 hours and thereafter treated with 50 μmol/L BOLD-100 for 24 hours and ROS levels assessed using the ROS-Glo H_2_O_2_ Assay Kit. **E,** Colorectal cancer cells were pretreated with 10 μmol/L CH-223191 (CH) for 3 hours and thereafter treated with 50 μmol/L BOLD-100 (B-100) for 24 hours and apoptosis assessed by WB for PARP (top) and Caspase 3/7 activity assay (bottom). Expression of pATR^T1989^, ATR and γH2AX was also determined. **F,** Schematic overview of proposed model. Oncogenic *BRAF* promotes BOLD-100 induced ATR/CHK1 activation by regulating replication stress through AhR/CYP1A1 and ROS. Coadministration of ATR inhibition (e.g., AZD6738) to BOLD-100 may promote replication fork collapse and apoptosis in *BRAF*MT colorectal cancer.

To further elucidate the mechanism by which BOLD-100 regulates ROS-mediated pATR/pCHK1 survival response, we focused on the genes identified in our 3 hours RNA-seq analysis as significantly upregulated following BOLD-100 treatment in VACO432 cells (Fig. 3B). Using the STRING database (string-db.org) to identify and visualize interactions, *CYP1A1*, *CYP1B1*, *LINC00551*, *TIPARP*, and *ALDH1A* upregulated genes formed a network around the *Aryl hydrocarbon Receptor* (*AhR*; Fig. 6A). We validated our RNA-seq results using real-time PCR, showing that *CYP1A1* mRNA levels were markedly upregulated as early as 3 hours following BOLD-100 treatment in *BRAF*MT cells [Fig. 6B (left); Supplementary Fig. S5D (top left)]. No changes in *CYP1A1* mRNA levels following BOLD-100 treatment were observed in the *BRAF*WT VT1 clone [Fig. 6B (right)]. Furthermore, treatment of *BRAF*MT colorectal cancer cells with vemurafenib significantly decreased BOLD-100-induced *CYP1A1* mRNA levels [Fig. 6C (left); Supplementary Fig. S5D (top middle)].

The AhR is a cytosolic, ligand-dependent transcription factor, playing a role in a variety of biological processes such as drug metabolism (32, 33). Activation of AhR is responsible for the increase in canonical cytochrome P450 metabolism enzymes, including CYP1A1, which is major source of ROS generation (34). Therefore, we evaluated the involvement of AhR in regulating BOLD-100-induced *CYP1A1* expression and ROS, using the AhR agonist tapinarof (11) and the AhR antagonist CH-223191 (9). Treatment with tapinarof resulted in marked increased *CYP1A1* mRNA expression in *BRAF*MT cells, but not in the *BRAF*WT clone (Supplementary Fig. S5E). Notably, treatment of *BRAF*MT colorectal cancer cells with CH-223191 significantly reduced basal and BOLD-100-induced *CYP1A1* mRNA levels and resulted in potent inhibition of ROS levels following BOLD-100 treatment [Fig. 6B–D; Supplementary Fig. S5D (bottom)]. In addition, BOLD-100-induced γH2AX and apoptosis were completely abrogated upon co-treatment with CH-223191 (Fig. 6E). Moreover, BOLD-100 increased *AhR* mRNA levels, and this was abrogated following preincubation with vemurafenib [Fig. 6C (right); Supplementary Fig. S5D (top right)]. Interestingly, basal AhR levels were higher in *the BRAF*MT VACO432 cells compared with its *BRAF*WT clone (Supplementary Fig. S5F). Taken together, these data would indicate that AhR directly regulates ATR/CHK1/γH2AX survival response in response to BOLD-100 treatment in *BRAF*MT colorectal cancer.

## Discussion

The phase III BEACON clinical trial made progress in the development of BRAF inhibitors by establishing combined encorafenib/cetuximab as a new standard of care for patients with *BRAFV*600E metastatic colorectal cancer who progressed to one or two previous lines of chemotherapy (3). However, not all patients respond to this combination, and some responses are short-lived (35). Identifying new therapeutic strategies to boost antitumor activity and improve survival of patients with *BRAF*MT colorectal cancer is paramount. BOLD-100 (formerly IT-139/NKP-1339/KP1339) is a first-in-class clinical-stage ruthenium-based small molecule drug and modulator of the UPR through selective inhibition of GRP78 (36). A phase I clinical trial of BOLD-100 in patients with advanced solid tumors showed that two patients with colorectal cancer demonstrated the largest reduction in target lesion (7). In this study, we addressed two major challenges facing the further clinical development of BOLD-100 in colorectal cancer: lack of predictive biomarkers to enable patient selection and emergence of acute resistance. Using a targeted TSG siRNA screen and mechanistic studies, we identified *caspase-8* loss as a mediator of resistance to BOLD-100, which is consistent with data from a previous study (37). This is the first study showing that the *BRAF* activating V600E mutation mediates response to BOLD-100 treatment in colorectal cancer.

Understanding which genes and pathways are complicit in the development of acute resistance to BOLD-100 allows the possibility of adopting therapeutic strategies to boost antitumor activity and prevent resistance. Using a systems biology approach, we found that BOLD-100 treatment increases reliance on the ATR-CHK1 pathway for survival of *BRAF*MT colorectal cancer cells. Ataxia telangiectasia and Rad3-related (ATR) is one of the central replication stress response kinases and plays a critical role in safeguarding genome integrity, preventing replication fork collapse, and favoring the repair of damaged forks (38). A number of previous studies reported the role of the replication stress response and ATR in mediating intrinsic resistance to chemotherapy and radiotherapy (39, 40). In the current study, we found that treatment with BOLD-100 resulted in acute increases in ATR/CHK1 phosphorylation, in particular in colorectal cancer cells with activated/oncogenic BRAF signaling. An oncogene-induced DNA replication stress model, in which oncogenes-induced genomic instability is because of DNA replication stress, has previously been described (41). Interestingly, our study also showed DNA_REPAIR as the top hallmark pathway deregulated in *BRAF*MT colorectal cancer cells (Fig. 1B).

CYP1A1 and CYP1B1 are extrahepatic P450 family members involved in the metabolism of drugs and xenobiotic molecules, which are a major source of ROS formation (42). ROS are well recognized as mediators of DNA damage (43). Mechanistically, we found that BOLD-100 acutely upregulates *CYP1A1* mRNA and ROS levels, particularly in colorectal cancer cells, with activated *BRAF* oncogene. Notably, treatment with the ROS scavenger NAC attenuated BOLD-100-induced ROS as well as ATR/CHK1 phosphorylation levels and resulted in robust decreases in BOLD-induced apoptosis in *BRAF*MT colorectal cancer cells. To our knowledge, this is the first evidence to link AhR-mediated ROS to ATR-CHK1 in the regulation of BOLD-100-resistance, in particular in *BRAF*MT colorectal cancer cells. The AhR is a cytoplasmic transcription factor that is well known for regulating xenobiotic metabolism. Canonical AhR signaling involves heterodimerization of ligand bound-AhR to the ARNT, which, in turn, induces the transcription of a number of its target genes, including CYP1A1. The major sources of AhR ligands include structurally diverse environmental/endogenous ligands (e.g., tryptophan catabolites) but also a number of exogenous ligands (e.g., benzoflavones; ref. 44). Mechanistically, our study showed that the AhR controls BOLD-100 induced CYP1A1 levels, ROS production, and ATR activation in *BRAF*MT colorectal cancer cells. Overall, our results suggest that oncogenic *BRAF* promotes BOLD-100 induced ATR/CHK1 activation by regulating replication stress through AhR/CYP1A1 and ROS (Fig. 6F).

The importance of ATR/CHK1 as mediator of acute resistance to BOLD-100 was demonstrated using a range of ATR inhibitors and several siRNA sequences targeting ATR. We found that ATR inhibition abrogates BOLD-100 induced ATR/CHK1 activation, leading to synergistic decreases in cell viability and colony formation, in addition to robust increases in apoptosis when ATR inhibition was combined with BOLD-100 in *BRAF*MT colorectal cancer cells. We propose that BOLD-100 synergizes with ATR inhibition by elevating replication stress through at least three mechanisms: first, by decreasing expressing of ribonucleotide reductase subunit M2 (RRM2), a rate-limiting enzyme in the synthesis of dNTP (Fig. 3C; Supplementary Fig. S3D; ref. 45). Second, by downregulation of other key enzymes (e.g., FEN1, FANC1) involved in DNA replication and repair (Fig. 3C; Supplementary Fig. S3D; refs. 46, 47). Third, by the induction of ROS, which in turn affect intracellular dNTP pools, by reducing ribonucleotide reductase activity and thereby replication fork velocity *in vitro* (48). Additionally, oxidized base lesions occurring from increased ROS activity can also induce DNA strand breaks and/or base modifications resulting in replication stress (49). Co-administration of ATR inhibition to BOLD-100 may therefore promote replication fork collapse and apoptosis in *BRAF*MT colorectal cancer (Fig. 6F).

Drugs targeting ATR (e.g., M6620) as monotherapy or combined with radio/chemotherapy are in early phase I/II clinical trial development (ClinicalTrials.gov). Based on promising results for BOLD-100 in patients with colorectal cancer in a phase I monotherapy trial (7), a phase Ib/2a clinical trial of BOLD-100 with FOLFOX is ongoing, with initial results showing good tolerability (50). To extend our *in vitro* findings, we assessed the therapeutic efficacy of combined BOLD-100 and AZD6738 in a *BRAF*MT xenograft model, showing supra-additive reductions in tumor growth with combined BOLD-100/AZD6738 treatment. Unexpectedly, this combination was less well tolerated in our *in vivo* strain, indicating that other ATR inhibitors (e.g., M6620), different treatment schedules, and doses will need to be explored *in vivo* before carrying this combination forward into a clinical trial.

In conclusion, we identified BRAF as a biomarker for response to BOLD-100 in colorectal cancer. We provide evidence that oncogenic *BRAF* controls replication stress and ATR/CHK1 activation following BOLD-100 treatment in an AhR/CYP1A1/ROS-dependent manner. From a cancer therapeutics perspective, the promising tumor growth inhibition observed in our xenograft study support the evaluation of BOLD-100 in combination with novel ATR inhibitors or other inhibitors of the replication stress response (e.g., CHK1 inhibitors) in clinical trials for patients with metastatic *BRAF*MT colorectal cancer.

## Supplementary Material

Supplementary Figure 1 Response of BRAFMT CRC cells to BOLD-100.

Supplementary Figure 2 Validation of the hits identified in the tumour suppressor genes (TSG) siRNA screen.

Supplementary Figure 3 Treatment with BOLD-100 rewires the signalling network of BRAFMT CRC cells.

Supplementary Figure 4 Treatment of BRAFMT CRC in vitro and mouse models with BOLD-100, AZD6738 or combination.

Supplementary Figure 5 BOLD-100 regulates apoptosis through BRAFMT-dependent AhR/CYP1A1/ROS/ATR axis activation.

Supplementary Mat & Methods Supplementary M & M

Table S1 Table S1: Antibodies

Table S2 Table S2: TSG siRNA screen

Table S3 Table S3: GSE59857 ssGSEA Hallmarks

Table S4 Table S4: Enrichr KEGG Pathways

Table S5 Table S5: BOLD-100 Compound Screen
