# Supplementary material for: Ruthenium Drug BOLD-100 Regulates BRAFMT Colorectal Cancer Cell Apoptosis through AhR/ROS/ATR Signaling Axis Modulation
Source: Mol Cancer Res. 2024 Jul 31;22(12):1088–101. doi: 10.1158/1541-7786.MCR-24-0151 (PMC7616621; doi:10.1158/1541-7786.MCR-24-0151)
Supplement: Supplementary Figure 1 — Response of BRAFMT CRC cells to BOLD-100. [file mcr-24-0151_supplementary_figure_1_suppsf1.pdf]

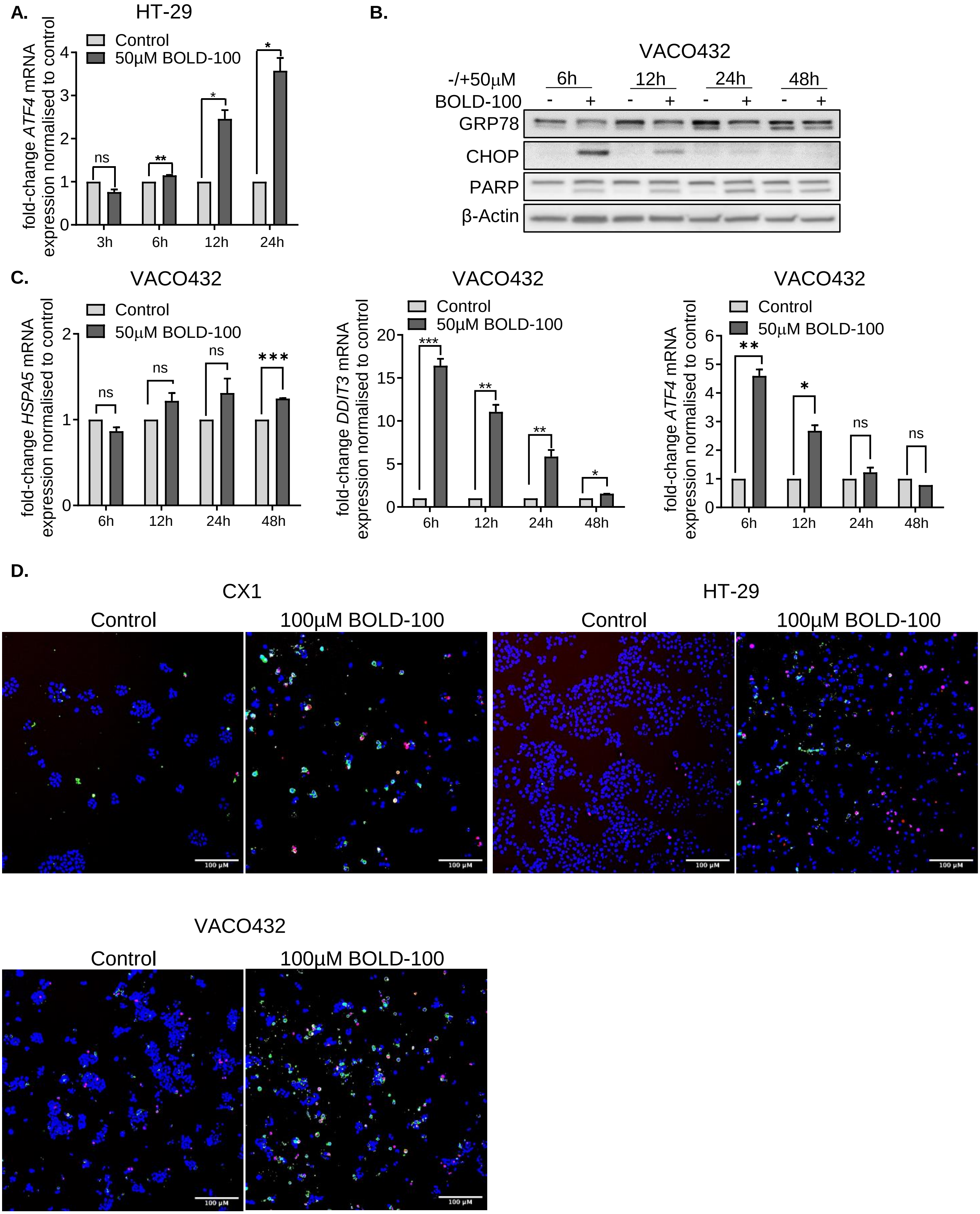
A.
HT-29
Control
50μM BOLD-100
fold-change ATF4 mRNA expression normalised to control
0
1
2
3
4
ns
**
*
*
3h
6h
12h
24h
B.
VACO432
-/+50μM BOLD-100
6h
12h
24h
48h
- + - + - + - +
GRP78
CHOP
PARP
β-Actin
C.
VACO432
Control
50μM BOLD-100
fold-change HSPA5 mRNA expression normalised to control
0
1
2
ns
ns
ns
***
6h
12h
24h
48h
VACO432
Control
50μM BOLD-100
fold-change DDIT3 mRNA expression normalised to control
0
5
10
15
20
***
**
**
*
6h
12h
24h
48h
VACO432
Control
50μM BOLD-100
fold-change ATF4 mRNA expression normalised to control
0
1
2
3
4
5
6
**
*
ns
ns
6h
12h
24h
48h
D.
CX1
Control
100μM BOLD-100
HT-29
Control
100μM BOLD-100
100 μM
VACO432
Control
100μM BOLD-100

D. Continued

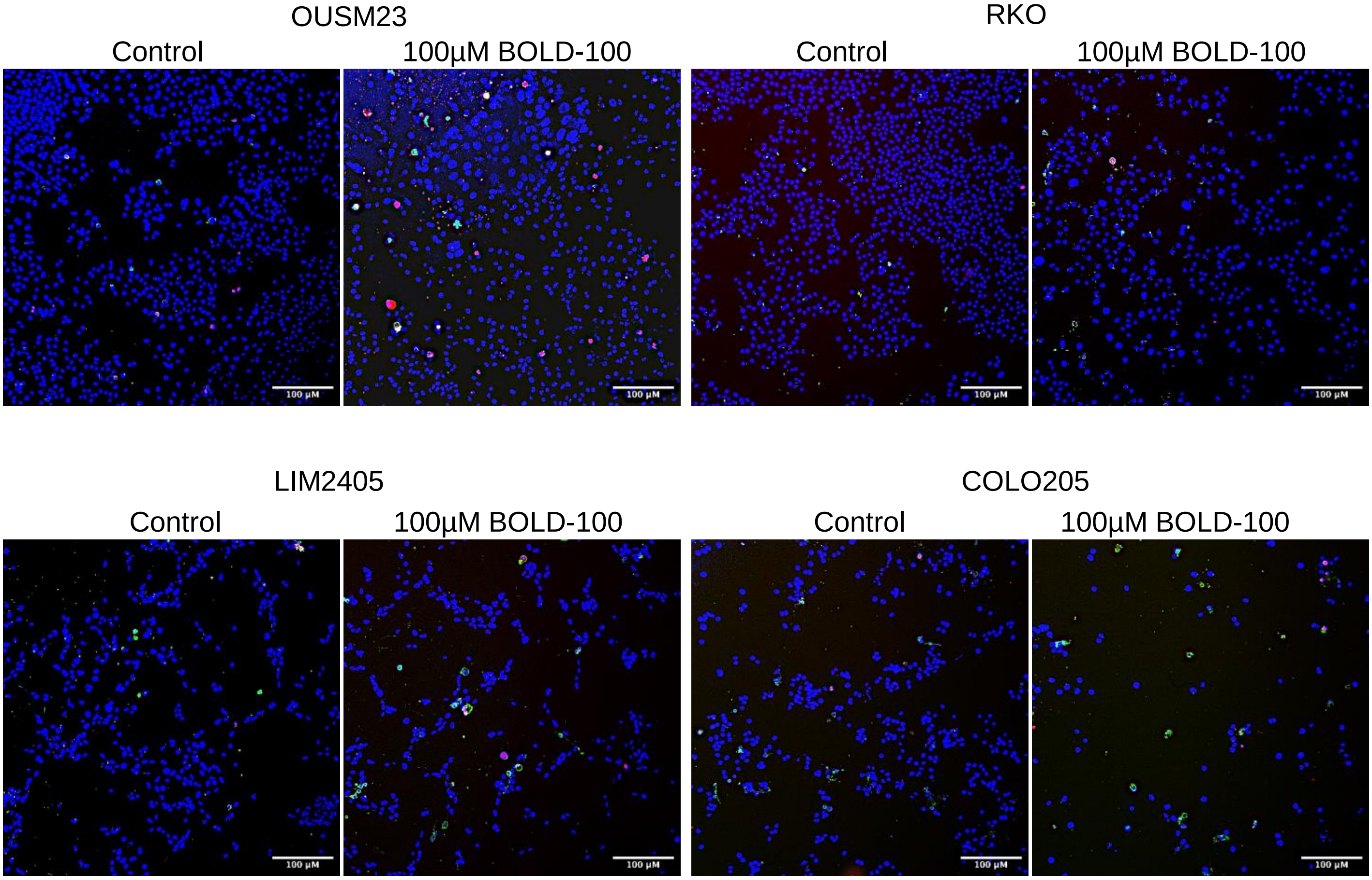

Supplementary figure 1. Response of *BRAF*MT CRC cells to BOLD-100. A. HT-29 cells were treated with BOLD-100 for the indicated times. *ATF4* mRNA expression levels were determined using RT-PCR. **B**. VACO432 cells were treated with BOLD-100 for the indicated times. GRP78, CHOP and PARP levels were determined by Western blotting (WB). **C.** VACO432 cells were treated with BOLD-100 for the indicated times. *HSPA5* (**Left**), *DDIT3* (**Middle**) and *ATF4* (**right**) mRNA was quantified using RT-PCR. Raw values were normalised to the expression of housekeeping genes *ACTB* and *GAPDH* and were analysed using the ΔΔCT method. mRNA levels presented are relative to untreated control. **D**. Representative images from data in **Fig. 1D right** are illustrated. *BRAF*MT CRC cells were treated with BOLD-100. Cells were stained with Hoechst (blue stain), AV (green) and PI (red).
