# Supplementary material for: Ruthenium Drug BOLD-100 Regulates BRAFMT Colorectal Cancer Cell Apoptosis through AhR/ROS/ATR Signaling Axis Modulation
Source: Mol Cancer Res. 2024 Jul 31;22(12):1088–101. doi: 10.1158/1541-7786.MCR-24-0151 (PMC7616621; doi:10.1158/1541-7786.MCR-24-0151)
Supplement: Supplementary Figure 2 — Validation of the hits identified in the tumour suppressor genes (TSG) siRNA screen. [file mcr-24-0151_supplementary_figure_2_suppsf2.pdf]

A.

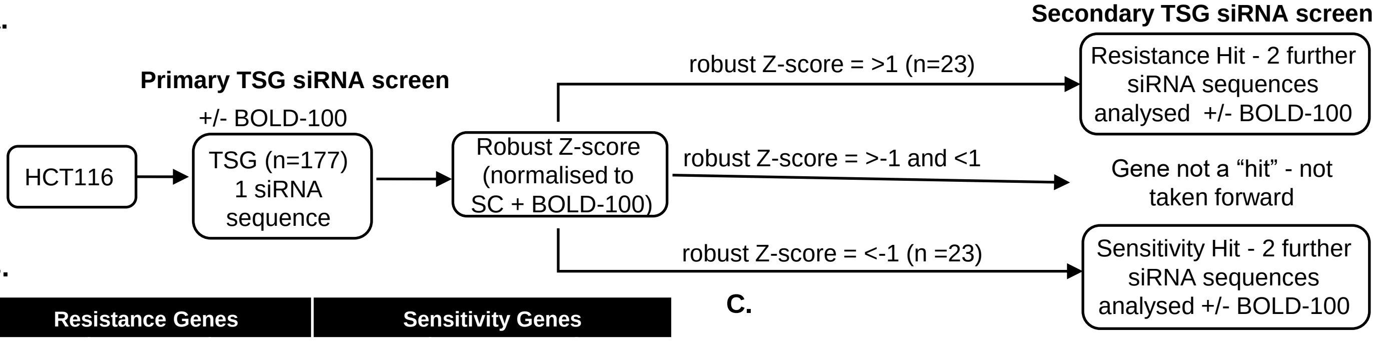

B.

Resistance Genes			Sensitivity Genes		
Gene	Mean r Z-score	SD	Gene	Mean r Z-score	SD
CASP8	2.2145	0.8332	ATR	-1.1594	0.1417
PTTG1IP	2.2008	0.3034	NPRL2	-1.4594	0.0835
ERCC1	2.1762	0.6919	TNFSF10	-1.7999	0.1554
RBBP7	2.1543	0.7055	RAD54L	-1.851	0.5046
PTTG2	1.9633	0.5633	E2F1	-1.8528	0.1004
RB1	1.8759	0.1788	SUFU	-2.0228	0.2094
TP53I11	1.7506	0.1344	JUNB	-2.1005	0.8693
GLTSCR1	1.7349	0.0976	RSPH14	-2.1483	0.234
FANCG	1.4852	0.417	GLTSCR2	-2.1874	0.4682
RB1CC1	1.4793	0.3478	MTUS1	-2.3731	1.3883
BLM	1.4432	0.0412	SACM1L	-2.4025	0.1742
APC	1.4359	0.272	CYB561D2	-2.4963	0.7159
WWOX	1.4323	0.4229	HSP90B1	-2.5187	0.0852
CDKN1A	1.34	0.3875	WTAP	-2.5385	0.3774
WT1	1.3304	0.2	DLC1	-2.5841	0.0221
TSG101	1.214	0.2023	NF1	-2.6724	0.1926
PALB2	1.2086	0.06	KSR2	-2.7291	0.4107
XPA	1.2039	0.1081	OVCA2	-2.7526	0.876
ZNF280B	1.2023	0.0113	GPS1	-2.7826	0.0744
ERAP1	1.1887	0.1383	MEN1	-2.9063	0.3811
VBP1	1.1492	0.0554	LZTS2	-2.9254	0.8563
TP53	1.1191	0.0533	DMBT1	-2.9432	0.08
TUSC3	1.1096	0.0508	TGFBR2	-3.788	0.2526

C.

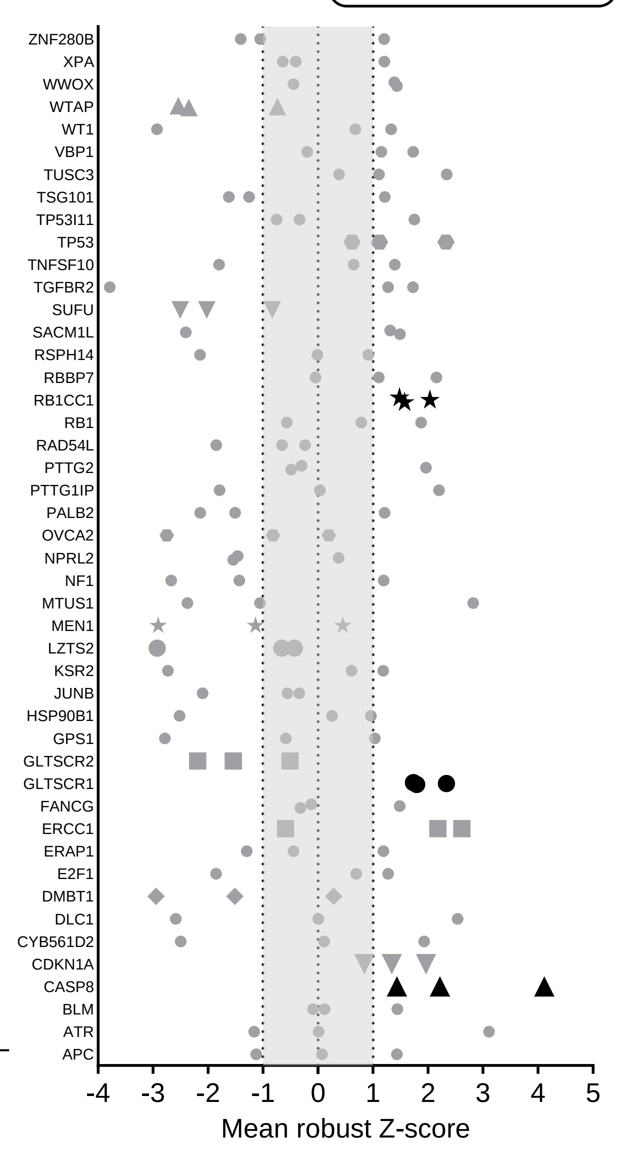

D.

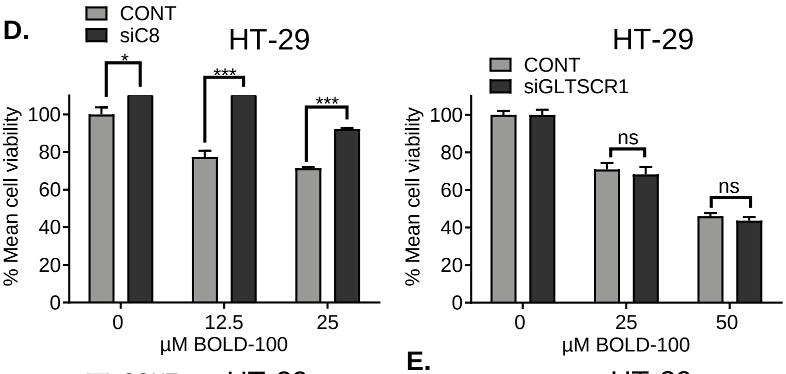

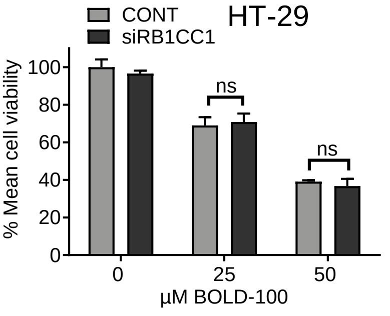

E.

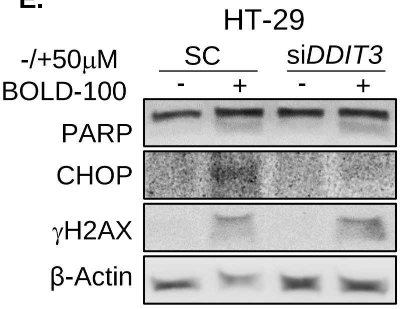

F.

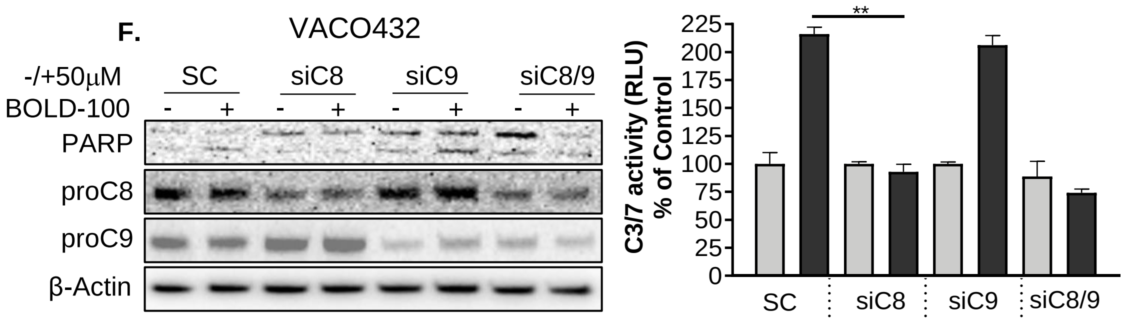

Supplementary figure 2. Validation of the hits identified in the tumour suppressor genes (TSG) siRNA screen. A. Schematic of the TSG siRNA screens in *KRAS*MT HCT116 cells to identify potential mediators of sensitivity and resistance to BOLD-100 treatment. HCT116 cells were reverse transfected with 1 siRNA targeting 177 TSG and 24h later treated with either DMSO control or 50µM BOLD-100 for 48h and cell viability was evaluated using the CTG assay. Positive scores indicate potential mediators of resistance to BOLD-100, while negative scores indicate mediators of sensitivity to BOLD-100. Robust Z-Scores were calculated and cut-off thresholds of +/-1 were applied to the data. This resulted in 46 potential hits, which were validated in a secondary screen using 2 independent siRNA sequences per gene. **B.** Table with positive hits from primary screen with mean rZ-score. **C.** Scatter plot showing robust Z-scores (rZ) for the 3 independent (1 used in primary screen, 2 in secondary screen) siRNA sequences for TSGs evaluated in the secondary siRNA screen in the HCT116 cells. Dashed lines indicate rZ scores = 1 and -1; cut-off thresholds of +/-1.0 were applied to the data. **D.** Hits with robust z-scores of >1.0 or <-1.0 for 3 independent siRNA sequences, were carried forward for validation studies. *BRAF*MT HT-29 cells were transfected with either 10nM SC or 10nM siRB1CC1 (pooled sequences Hs_RB1CC1_1, Hs_RB1CC1_6, Hs_RB1CC1_7), 10nM siGLTSCR1 (pooled sequences Hs_GLTSCR1_1, Hs_GLTSCR1_7, Hs_GLTSCR1_8) or 10nM siCaspase 8 (pooled sequences Hs_CASP8_1, Hs_CASP8_7, Hs_CASP8_12) for 24h and then treated with the indicated doses of BOLD-100 for 48h. CTG assay was used to evaluate cell viability, which is presented relative to SC control. **E**. WB analysis of PARP, CHOP and γH2AX expression in HT-29 cells, following transfection with SC or siDDIT3 for 24h, and treatment with BOLD-100 for an additional 48h**. F.** *BRAF*MT VACO432 cells were transfected with 10nM *C8* or/and *C9* siRNA for 24h and thereafter treated with BOLD-100 for 24h. Apoptosis was assessed by WB analysis for PARP (**left**) and caspase-3/7 activity (**right**). Expression of pro-caspase-8 and pro-caspase-9 are also shown.
