# Supplementary material for: Ruthenium Drug BOLD-100 Regulates BRAFMT Colorectal Cancer Cell Apoptosis through AhR/ROS/ATR Signaling Axis Modulation
Source: Mol Cancer Res. 2024 Jul 31;22(12):1088–101. doi: 10.1158/1541-7786.MCR-24-0151 (PMC7616621; doi:10.1158/1541-7786.MCR-24-0151)
Supplement: Supplementary Figure 3 — Treatment with BOLD-100 rewires the signalling network of BRAFMT CRC cells. [file mcr-24-0151_supplementary_figure_3_suppsf3.pdf]

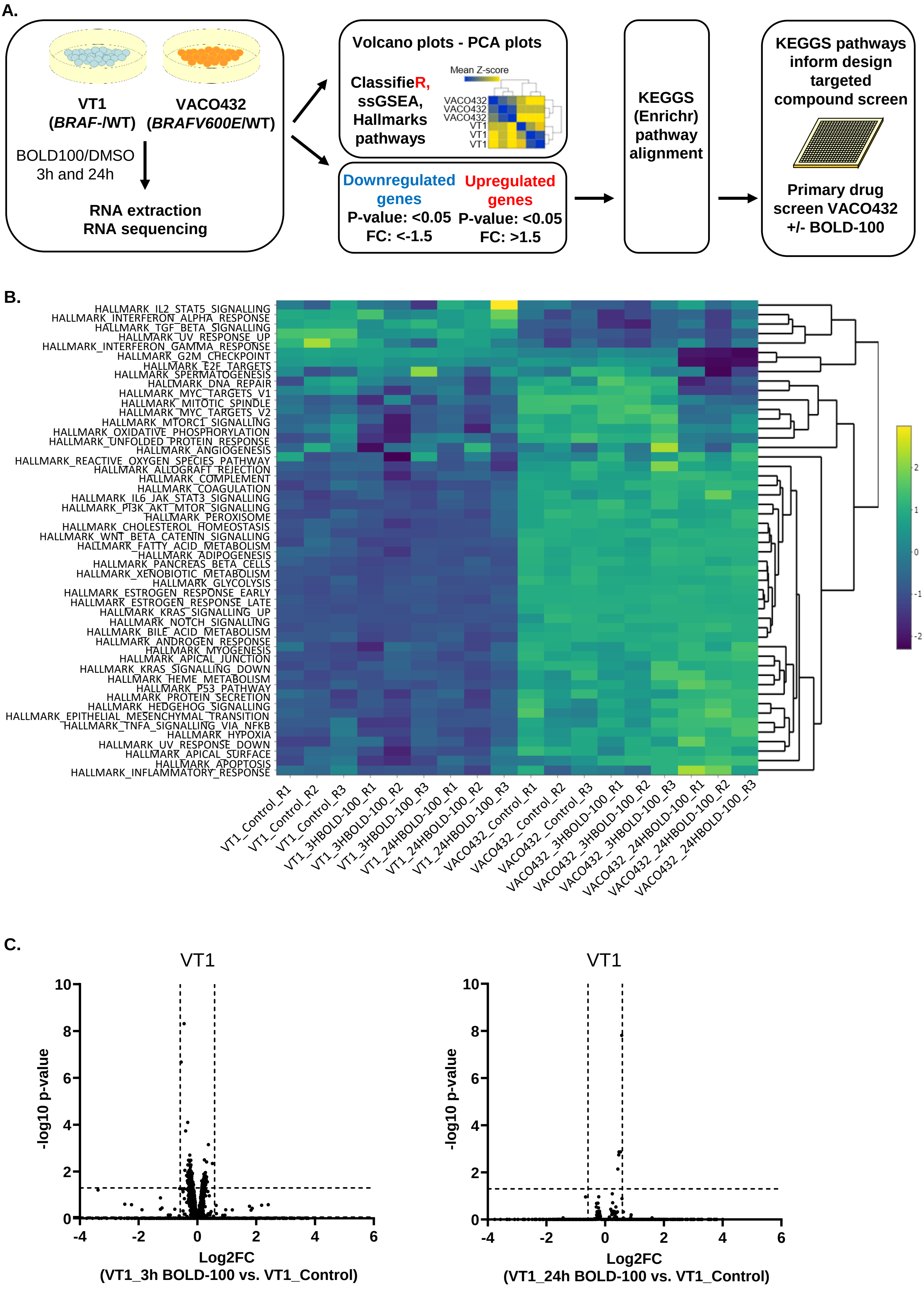
A.
VT1
(BRAF-/WT)
VACO432
(BRAFV600E/WT)
BOLD100/DMSO
3h and 24h
RNA extraction
RNA sequencing
Volcano plots - PCA plots
ClassifieR,
ssGSEA,
Hallmarks
pathways
Mean Z-score
VACO432
VACO432
VACO432
VT1
VT1
VT1
Downregulated genes
P-value: <0.05
FC: <-1.5
Upregulated genes
P-value: <0.05
FC: >1.5
KEGGS
(Enrichr)
pathway
alignment
KEGGS pathways
inform design
targeted
compound screen
Primary drug
screen VACO432
+/- BOLD-100
B.
HALLMARK_IL2_STAT5_SIGNALLING
HALLMARK_INTERFERON_ALPHA_RESPONSE
HALLMARK_TGF_BETA_SIGNALLING
HALLMARK_UV_RESPONSE_UP
HALLMARK_INTERFERON_GAMMA_RESPONSE
HALLMARK_G2M_CHECKPOINT
HALLMARK_E2F_TARGETS
HALLMARK_SPERMATOGENESIS
HALLMARK_DNA_REPAIR
HALLMARK_MYC_TARGETS_V1
HALLMARK_MITOTIC_SPINDLE
HALLMARK_MYC_TARGETS_V2
HALLMARK_MTORC1_SIGNALLING
HALLMARK_OXIDATIVE_PHOSPHORYLATION
HALLMARK_UNFOLDED_PROTEIN_RESPONSE
HALLMARK_ANGIOGENESIS
HALLMARK_REACTIVE_OXYGEN_SPECIES_PATHWAY
HALLMARK_ALLOGRAFT_REJECTION
HALLMARK_COMPLEMENT
HALLMARK_COAGULATION
HALLMARK_IL6_JAK_STAT3_SIGNALLING
HALLMARK_PI3K_AKT_MTOR_SIGNALLING
HALLMARK_PEROXISOME
HALLMARK_CHOLESTEROL_HOMEOSTASIS
HALLMARK_WNT_BETA_CATENIN_SIGNALLING
HALLMARK_FATTY_ACID_METABOLISM
HALLMARK_ADIPOGENESIS
HALLMARK_PANCREAS_BETA_CELLS
HALLMARK_XENOBIOTIC_METABOLISM
HALLMARK_GLYCOLYSIS
HALLMARK_ESTROGEN_RESPONSE_EARLY
HALLMARK_ESTROGEN_RESPONSE_LATE
HALLMARK_KRAS_SIGNALLING_UP
HALLMARK_NOTCH_SIGNALLING
HALLMARK_BILE_ACID_METABOLISM
HALLMARK_ANDROGEN_RESPONSE
HALLMARK_MYOGENESIS
HALLMARK_APICAL_JUNCTION
HALLMARK_KRAS_SIGNALLING_DOWN
HALLMARK_HEME_METABOLISM
HALLMARK_P53_PATHWAY
HALLMARK_PROTEIN_SECRETION
HALLMARK_HEDGEHOG_SIGNALLING
HALLMARK_EPITHELIAL_MESENCHYMAL_TRANSITION
HALLMARK_TNFA_SIGNALLING_VIA_NFKB
HALLMARK_HYPOXIA
HALLMARK_UV_RESPONSE_DOWN
HALLMARK_APICAL_SURFACE
HALLMARK_APOPTOSIS
HALLMARK_INFLAMMATORY_RESPONSE
VT1_Control_R1
VT1_Control_R2
VT1_Control_R3
VT1_3HBOLD-100_R1
VT1_3HBOLD-100_R2
VT1_3HBOLD-100_R3
VT1_24HBOLD-100_R1
VT1_24HBOLD-100_R2
VT1_24HBOLD-100_R3
VACO432_Control_R1
VACO432_Control_R2
VACO432_Control_R3
VACO432_3HBOLD-100_R1
VACO432_3HBOLD-100_R2
VACO432_3HBOLD-100_R3
VACO432_24HBOLD-100_R1
VACO432_24HBOLD-100_R2
VACO432_24HBOLD-100_R3
2
1
0
-1
-2
C.
VT1
-log10 p-value
Log2FC
(VT1_3h BOLD-100 vs. VT1_Control)
VT1
-log10 p-value
Log2FC
(VT1_24h BOLD-100 vs. VT1_Control)

D.

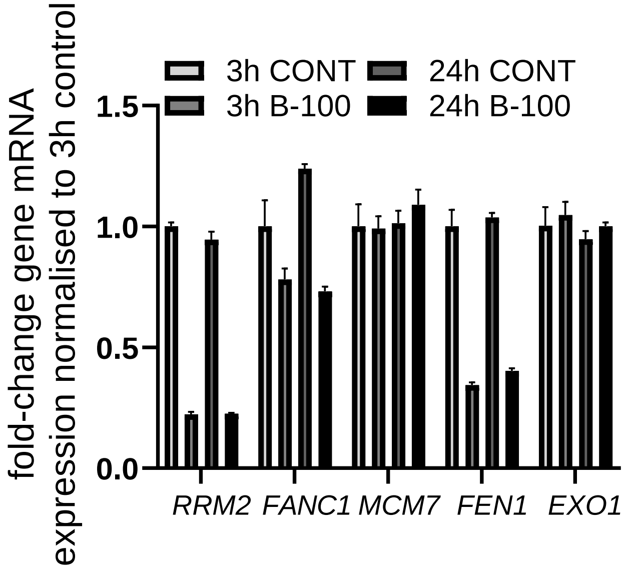
3h CONT
3h B-100
24h CONT
24h B-100
fold-change gene mRNA expression normalised to 3h control
1.5
1.0
0.5
0.0
RRM2
FANC1
MCM7
FEN1
EXO1

E.

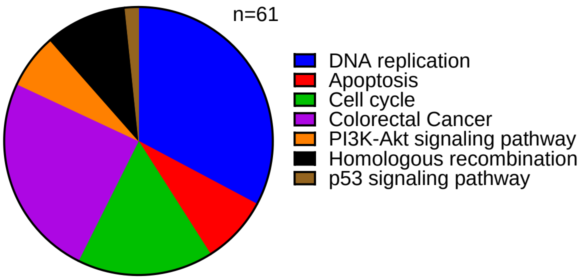
n=61
DNA replication
Apoptosis
Cell cycle
Colorectal Cancer
PI3K-Akt signaling pathway
Homologous recombination
p53 signaling pathway

F.

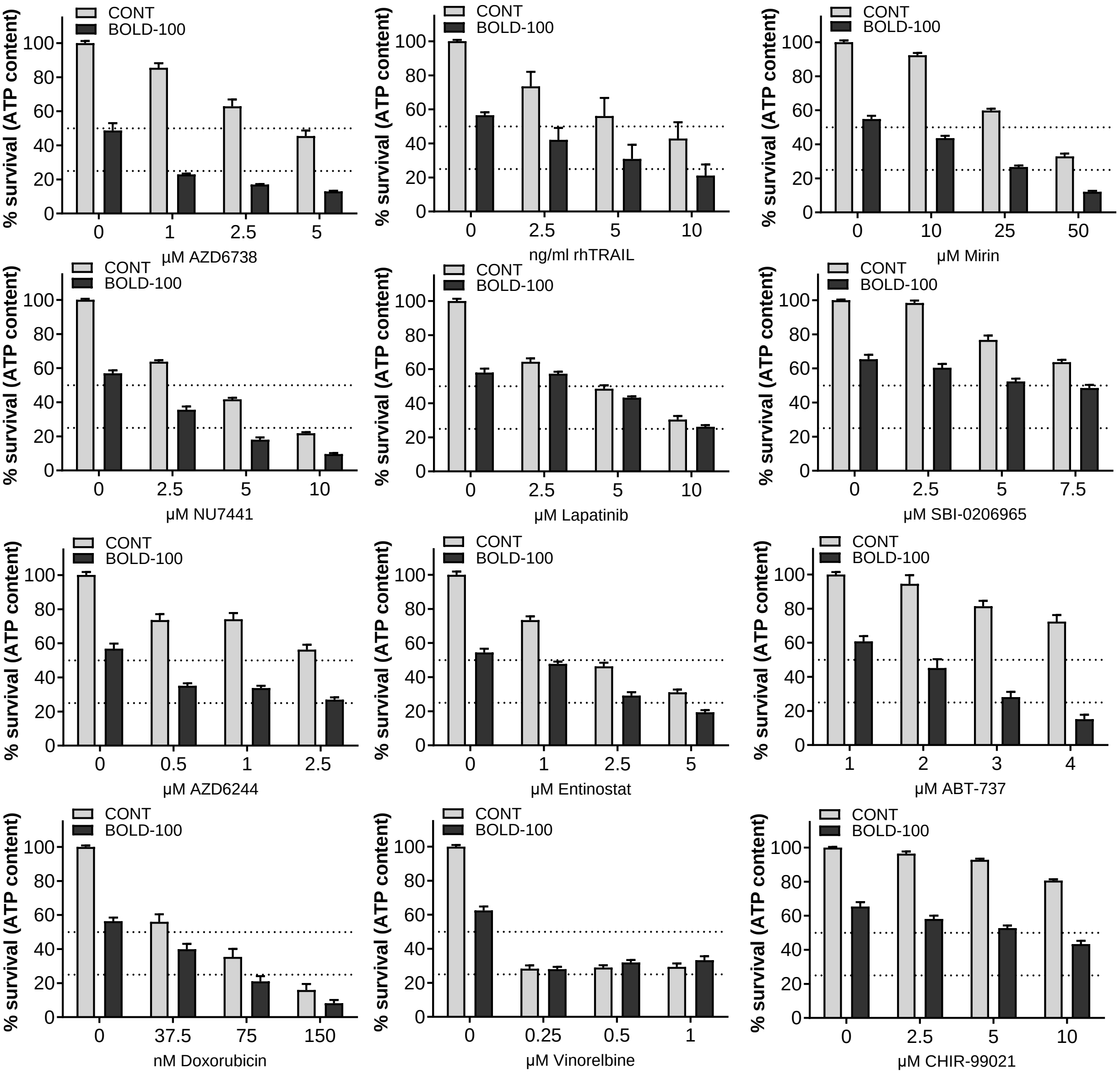
CONT
BOLD-100
% survival (ATP content)
0 20 40 60 80 100
0 1 2.5 5
µM AZD6738
0 2.5 5 10
ng/ml rhTRAIL
0 10 25 50
µM Mirin
0 2.5 5 10
µM NU7441
0 2.5 5 10
µM Lapatinib
0 2.5 5 7.5
µM SBI-0206965
0 0.5 1 2.5
µM AZD6244
0 1 2.5 5
µM Entinostat
1 2 3 4
µM ABT-737
0 37.5 75 150
nM Doxorubicin
0 0.25 0.5 1
µM Vinorelbine
0 2.5 5 10
µM CHIR-99021

Supplementary figure 3. Treatment with BOLD-100 rewires the signalling network of *BRAF*MT CRC cells. A. Schematic of the experimental pipeline and bioinformatics analysis to comprehensively map adaptive signalling following BOLD-100 in *BRAF*MT CRC. *BRAF*MT VACO432 CRC cells, and its isogenic *BRAF*WT VT1 clone were treated with 24µM BOLD-100 for the indicated times. Total RNA was extracted, library preparation performed and RNA sequencing conducted. Input data for the Volcano plots, PCA plots and enrichment analysis (ssGSEA) on 50 hallmark pathways (22) was the unfiltered gene list (N= 35468). Differential expression analysis was performed using the DESeq2 package. A cut-off threshold of fold-change >1.5 or <-1.5, and adjusted p-value<0.05 was applied to the gene lists. KEGGS pathway analysis was performed with Enrichr (BMC Bioinformatics 2013; 14: 128) using differential expressed genelists for VACO432 cells treated for 3h (n=5) and 24h (n=487) with BOLD-100. Outputs from this analysis are significant differentially expressed KEGG pathways. A focused drug screen was performed to identify compounds that could effectively suppress viability of *BRAF*MT CRC cells when combined with BOLD-100. A drug library targeting the top druggable KEGGS pathways was used. **B.** Raw gene counts were uploaded to the classifieR (BMC Bioinformatics 2022; 23: 114), and ssGSEA pathway analysis was performed. Two-dimensional hierarchical clustering analysis of hallmarks pathways that were induced or repressed following treatment of VACO432 and VT1 cells with BOLD-100. **C.** Volcano plots show the up- and downregulated genes following BOLD-100 treatment in VT1 cells at the indicated time-points. Dashed lines on the x and y-axis indicate log_2ratio of 0.58/-0.58, and $-log_{10}$ p-value=1.3, respectively. **D.** VACO432 cells were treated for indicated times with 50µM BOLD-100. *RRM2, FANC1, MCM7, FEN1, EXO1* mRNA were quantified using RT-PCR. Raw values were normalised to the expression of housekeeping genes *ACTB* and *GAPDH* and were analysed using the ΔΔCT method. mRNA levels presented are relative to SC. **E.** Pie chart of KEGG pathways targeted by drugs included in the primary compound screen. **F**. Absolute cell viability for positive hits from primary drug screen is shown. Dashed line indicates 50% and 25% cell viability. Mean of 3 independent experiments is shown.
