# Supplementary material for: Ruthenium Drug BOLD-100 Regulates BRAFMT Colorectal Cancer Cell Apoptosis through AhR/ROS/ATR Signaling Axis Modulation
Source: Mol Cancer Res. 2024 Jul 31;22(12):1088–101. doi: 10.1158/1541-7786.MCR-24-0151 (PMC7616621; doi:10.1158/1541-7786.MCR-24-0151)
Supplement: Supplementary Figure 4 — Treatment of BRAFMT CRC in vitro and mouse models with BOLD-100, AZD6738 or combination. [file mcr-24-0151_supplementary_figure_4_suppsf4.pdf]

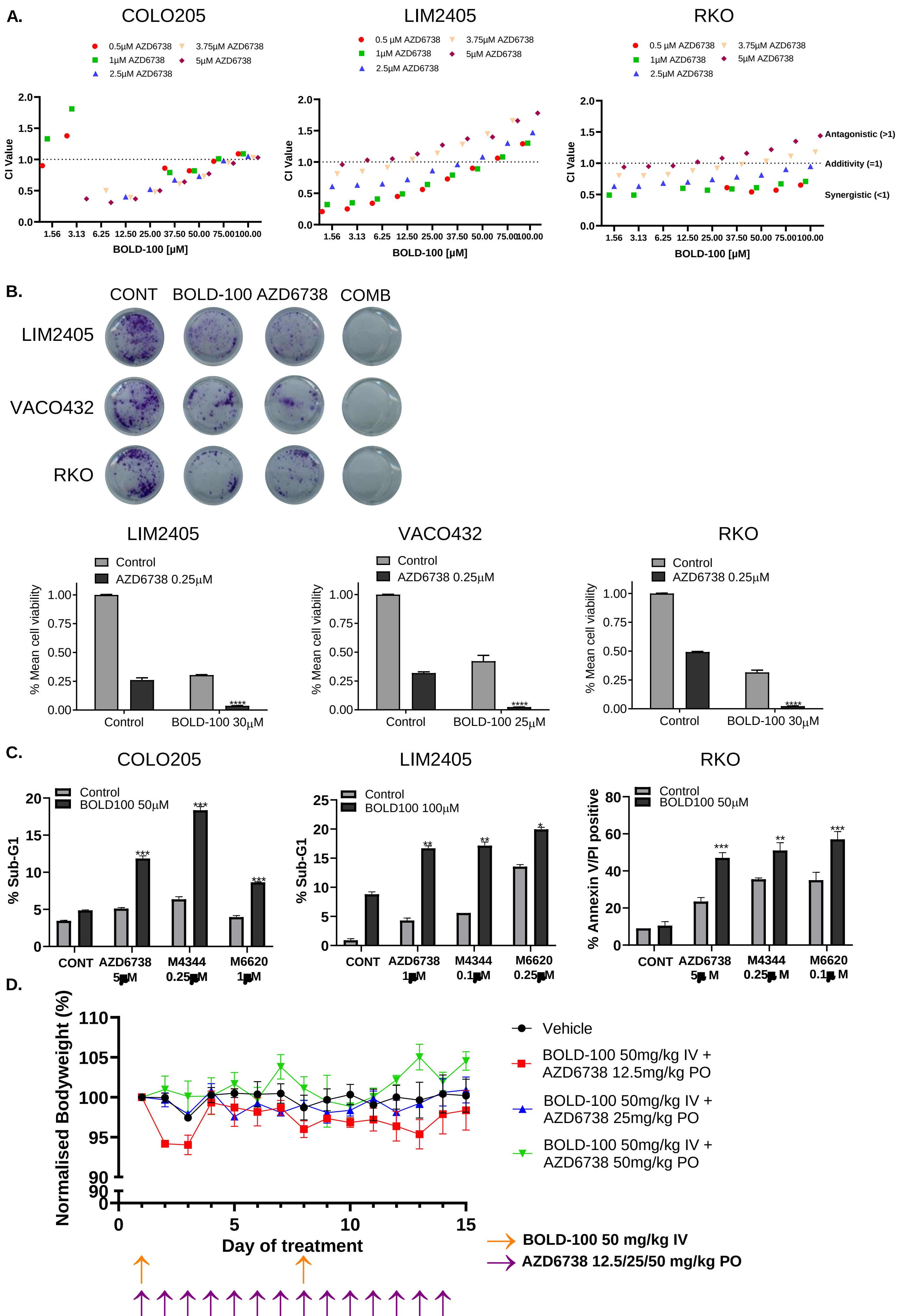

A.
COLO205
LIM2405
RKO
0.5μM AZD6738
1μM AZD6738
2.5μM AZD6738
3.75μM AZD6738
5μM AZD6738
CI Value
BOLD-100 [μM]
1.56 3.13 6.25 12.50 25.00 37.50 50.00 75.00 100.00
Antagonistic (>1)
Additivity (=1)
Synergistic (<1)
B.
CONT
BOLD-100
AZD6738
COMB
LIM2405
VACO432
RKO
Control
AZD6738 0.25μM
% Mean cell viability
BOLD-100 30μM
BOLD-100 25μM
C.
COLO205
LIM2405
RKO
Control
BOLD100 50μM
BOLD100 100μM
% Sub-G1
% Annexin V/PI positive
CONT
AZD6738 5μM
M4344 0.25μM
M6620 1μM
AZD6738 1μM
M4344 0.1μM
M6620 0.25μM
M6620 0.1μM
D.
Normalised Bodyweight (%)
Day of treatment
Vehicle
BOLD-100 50mg/kg IV + AZD6738 12.5mg/kg PO
BOLD-100 50mg/kg IV + AZD6738 25mg/kg PO
BOLD-100 50mg/kg IV + AZD6738 50mg/kg PO
BOLD-100 50 mg/kg IV
AZD6738 12.5/25/50 mg/kg PO

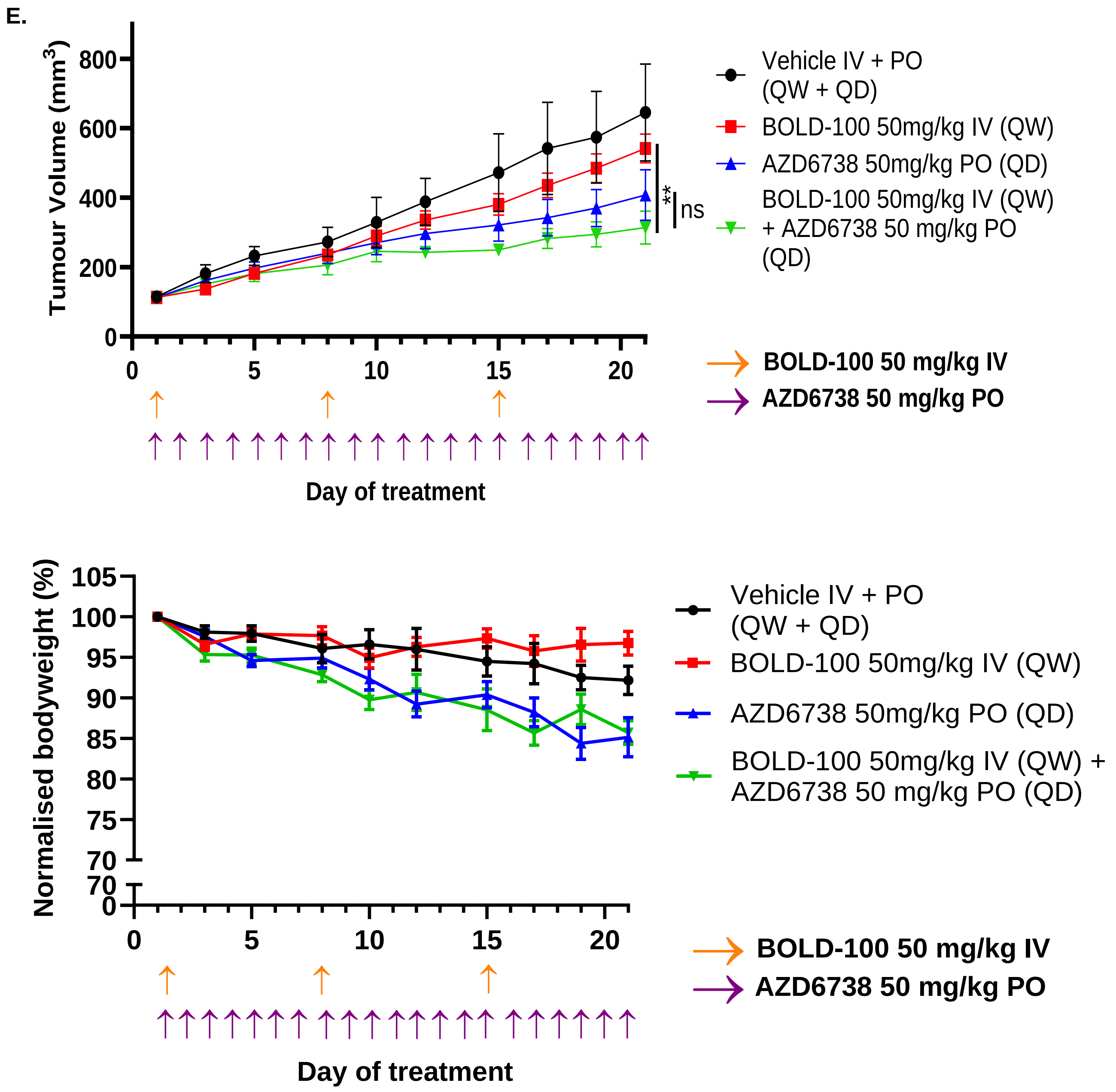


**Supplementary figure 4. Treatment of *BRAF*MT CRC *in vitro* and mouse models with BOLD-100, AZD6738 or combination. A.** CTG cell viability assays in *BRAF*MT CRC cells co-treated with BOLD-100 and ATR inhibitor AZD6738 for 72h. CI values were calculated to evaluate the nature of interaction. **B.** Clonogenic survival assays at day 14 in LIM2405, VACO432 and RKO cells treated with 30µM or 25µM BOLD-100 and/or 0.25µM AZD6738. Cells were fixed with methanol and stained with 0.01% crystal violet for 5 minutes at room temperature. Stained cells were reabsorbed in ethanol supplemented with 0.2 mol/l sodium citrate (1:1 dilution). Viability was determined by measuring the absorbance at 570 nm, and presented in a bar graph. **C.** COLO205, LIM2405 and RKO cells were co-treated with BOLD-100 and ATR inhibitors (AZD6738, M4344, M6620) for 48h and apoptosis was assessed using PI or Annexin V/PI staining. The graph indicates the percentage of positive stained cells. **D.** Normalised weights of non-tumour-bearing BALB/c Nude mice following 2 weeks treatment with combined BOLD-100/AZD6738. Dose levels and days of treatments are indicated on the graphs. The concentration of AZD6738 started at 12.5mg/kg and increased to 25mg/kg and 50mg/kg when tolerability was observed. Values shown are mean ±SEM; n=3 for all treatment groups. The average bodyweight of the BOLD-100 50mg/kg + AZD6738 12.5mg/kg, AZD6738 25mg/kg and AZD6738 50mg/kg treated animals on day 15 was 98.4%, 100.9% and 104.6% respectively of the initial weight. **E. Upper:** Growth rate of VACO432 xenografts in BALB/c Nude mice treated with vehicle, BOLD-100, AZD6738 or BOLD-100 in combination with AZD6738. **Lower:** Normalised weights of VACO432 xenografts in BALB/c Nude mice following 3 weeks treatment with BOLD-100, AZD6738 or BOLD-100 with AZD6738. Values shown are mean ±SEM. The average bodyweight of the Vehicle, BOLD-100 50mg/kg, AZD6738 50mg/kg and BOLD-100/AZD6738 treated animals on day 21 was 92.15%, 96.75%, 85.11% and 85.71% respectively of the initial weight.
