# Supplementary material for: Ruthenium Drug BOLD-100 Regulates BRAFMT Colorectal Cancer Cell Apoptosis through AhR/ROS/ATR Signaling Axis Modulation
Source: Mol Cancer Res. 2024 Jul 31;22(12):1088–101. doi: 10.1158/1541-7786.MCR-24-0151 (PMC7616621; doi:10.1158/1541-7786.MCR-24-0151)
Supplement: Supplementary Figure 5 — BOLD-100 regulates apoptosis through BRAFMT-dependent AhR/CYP1A1/ROS/ATR axis activation. [file mcr-24-0151_supplementary_figure_5_suppsf5.pdf]

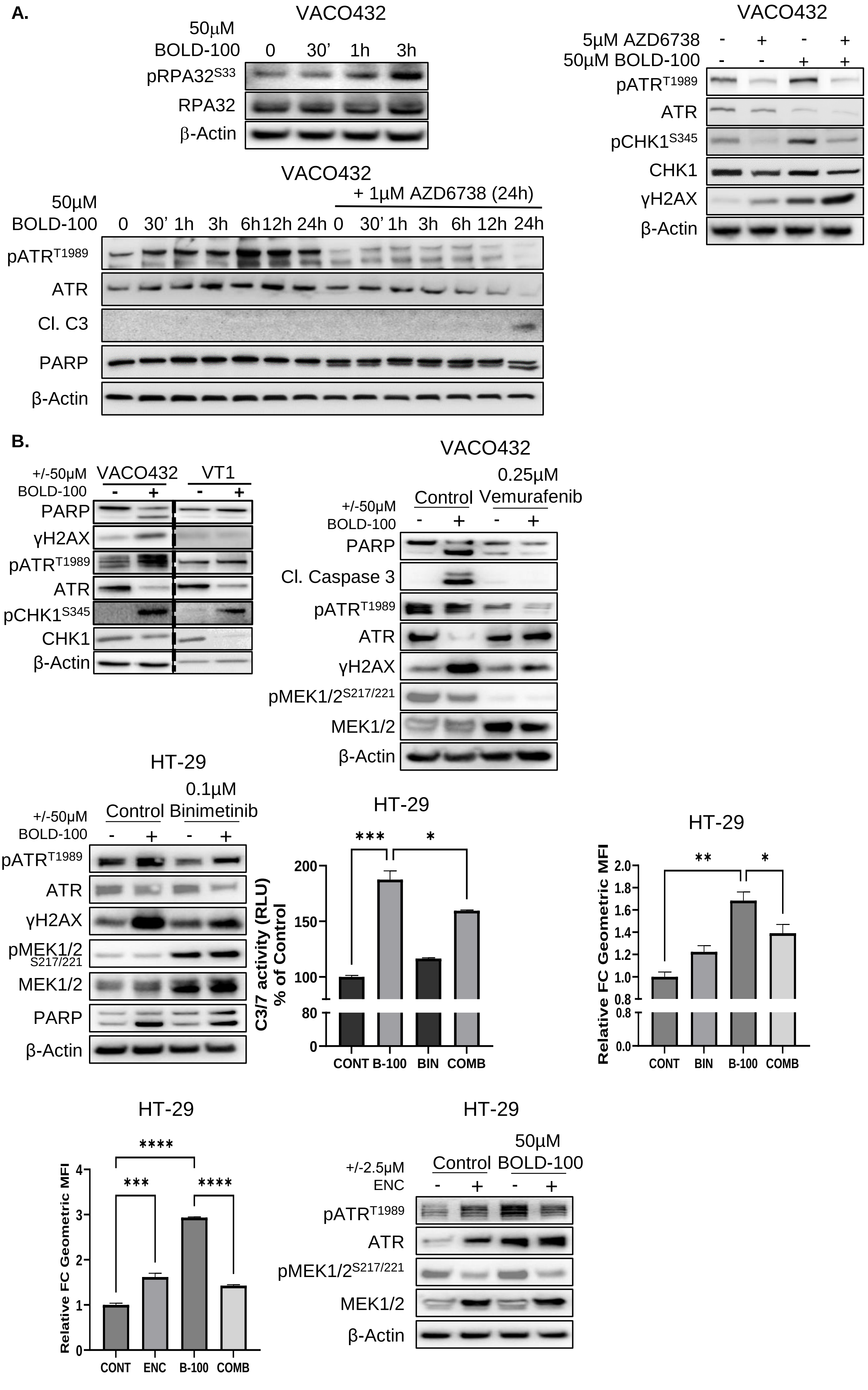
A.
VACO432
50μM BOLD-100
0
30'
1h
3h
pRPA32S33
RPA32
β-Actin
VACO432
5μM AZD6738
50μM BOLD-100
pATRT1989
ATR
pCHK1S345
CHK1
γH2AX
β-Actin
VACO432
+ 1μM AZD6738 (24h)
50μM BOLD-100
0 30' 1h 3h 6h 12h 24h 0 30' 1h 3h 6h 12h 24h
pATRT1989
ATR
Cl. C3
PARP
β-Actin
B.
+/-50μM BOLD-100
VACO432
VT1
PARP
γH2AX
pATRT1989
ATR
pCHK1S345
CHK1
β-Actin
VACO432
Control
0.25μM Vemurafenib
+/-50μM BOLD-100
PARP
Cl. Caspase 3
pATRT1989
ATR
γH2AX
pMEK1/2S217/221
MEK1/2
β-Actin
HT-29
Control
0.1μM Binimetinib
+/-50μM BOLD-100
pATRT1989
ATR
γH2AX
pMEK1/2 S217/221
MEK1/2
PARP
β-Actin
HT-29
C3/7 activity (RLU) % of Control
CONT
B-100
BIN
COMB
HT-29
Relative FC Geometric MFI
CONT
BIN
B-100
COMB
HT-29
Relative FC Geometric MFI
CONT
ENC
B-100
COMB
HT-29
+/-2.5μM ENC
Control
50μM BOLD-100
pATRT1989
ATR
pMEK1/2S217/221
MEK1/2
β-Actin

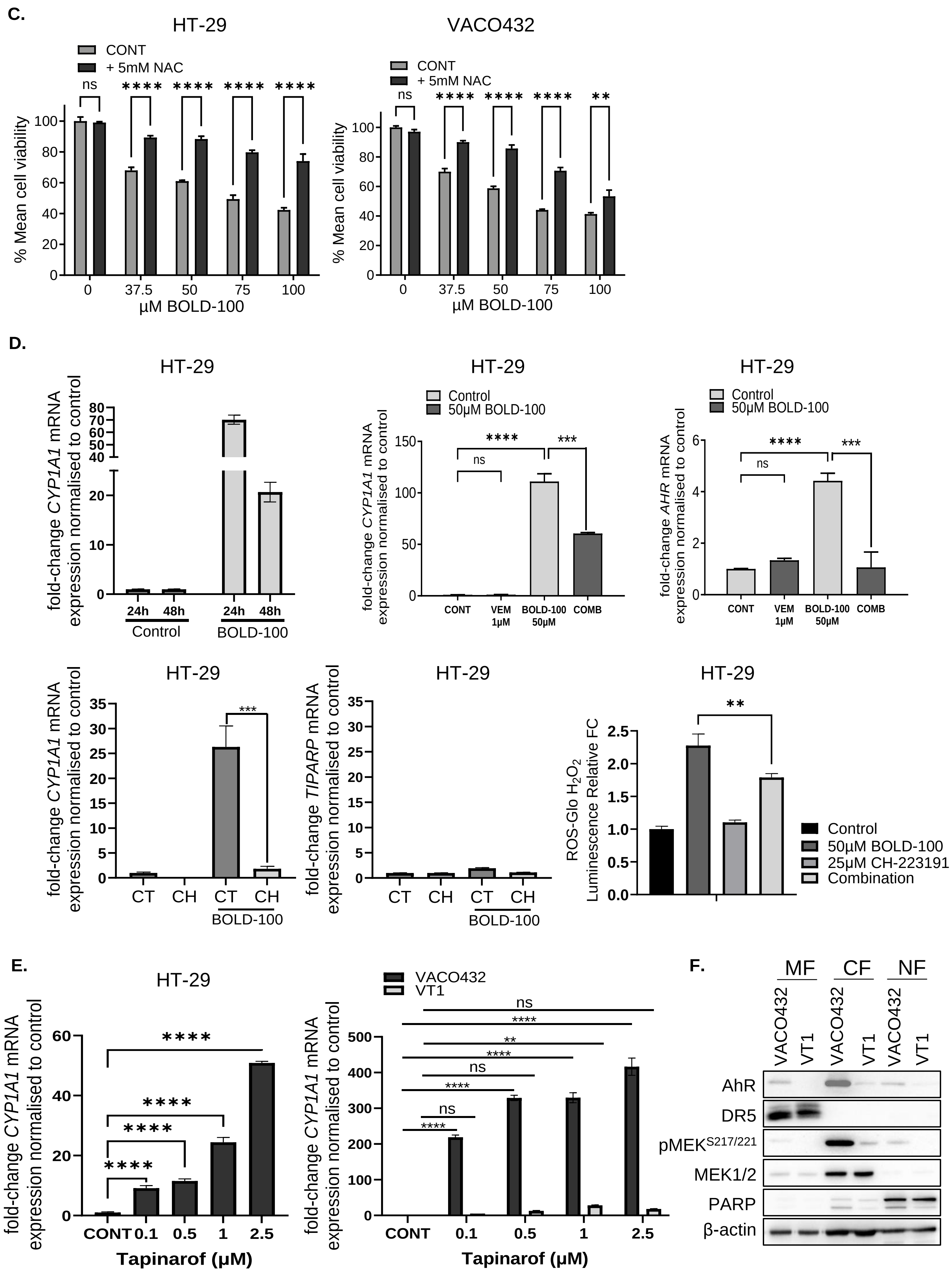
C.
HT-29
CONT
+ 5mM NAC
ns
****
% Mean cell viability
µM BOLD-100
0
37.5
50
75
100
VACO432
**
D.
HT-29
fold-change CYP1A1 mRNA expression normalised to control
24h
48h
Control
BOLD-100
Control
50µM BOLD-100
***
CONT
VEM 1µM
BOLD-100 50µM
COMB
fold-change AHR mRNA expression normalised to control
CT
CH
fold-change TIPARP mRNA expression normalised to control
ROS-Glo H_2O_2 Luminescence Relative FC
Control
50µM BOLD-100
25µM CH-223191
Combination
E.
HT-29
Tapinarof (µM)
CONT
0.1
0.5
1
2.5
VACO432
VT1
F.
MF
CF
NF
VACO432
VT1
AhR
DR5
pMEK[S217/221]
MEK1/2
PARP
β-actin

Supplementary figure 5. BOLD-100 regulates apoptosis through *BRAF*MT-dependent AhR/CYP1A1/ROS/ATR axis activation. A. Upper Left: VACO432 cells were treated with BOLD-100 for the indicated times. pRPA32^{S33} and RPA32 levels were determined by WB. **Lower left:** VACO432 cells were pre-treated with AZD6738 for 24h and thereafter treated with BOLD-100 for the indicated times. pATR^{T1989}, ATR, cleaved caspase 3 (Cl. C3) and PARP levels were determined by WB. **Upper right:** VACO432 cells were co-treated with AZD6738 and BOLD-100 for 24h and γH2AX, pATR^{T1989}, ATR, pCKH1^{S345} and CHK1 levels determined by WB. **B. Upper Left:** VACO432 and VT1 cells were treated with BOLD-100 for 6h and PARP, γH2AX, pATR^{T1989}, ATR, pCKH1^{S345} and CHK1 levels were determined by WB. **Upper right:** VACO432 cells were pre-incubated with Vemurafenib for 3h and thereafter treated with 50µM BOLD-100 for 24h and PARP, cleaved caspase-3, pATR^{T1989}, ATR, γH2AX, pMEK1/2$^{S217/221}$ and MEK expression determined by WB. **Middle Left:** HT-29 cells were pre-incubated with Binimetinib for 3h and thereafter treated with 50µM BOLD-100 for 24h and pATR^{T1989}, ATR, γH2AX, pMEK1/2$^{S217/221}$, MEK, PARP determined by WB. Apoptosis was assessed by caspase-3/7 activity assay. **Middle right:** ROS detection by flow cytometry in HT-29 cells, pre-incubated with 0.1µM Binimetinib (BIN) for 3h, followed by 24h treatment with 50µM BOLD-100. MFI = mean fluorescence intensity. **Lower Left:** ROS detection by flow cytometry in HT-29 cells, pre-incubated with 2.5µM Encorafenib (ENC) for 3h, followed by 24h treatment with 50µM BOLD-100. **Lower right:** HT-29 cells were pre-incubated with Encorafenib for 3h and thereafter treated with 50µM BOLD-100 for 24h and pATR^{T1989}, ATR, pMEK1/2$^{S217/221}$ and MEK expression determined by WB. **C.** Cells were pre-incubated with N-Acetyl-L-Cysteine (NAC) for 6h and thereafter treated with BOLD-100 for 48h and cell viability determined using MTT assays. **D. Upper, left:** HT-29 cells were treated for indicated times with 50µM BOLD-100. *CYP1A1* mRNA was quantified using RT-PCR. **Upper, middle, right:** HT-29 cells were pre-incubated with Vemurafenib (VEM) for 3h followed by BOLD-100 treatment for 24h and *CYP1A1* and *AHR* mRNA were quantified using RT-PCR. **Lower: Left and middle:** HT-29 cells were pre-treated with 10µM CH-223191 (CH) for 3h and thereafter treated with 50µM BOLD-100 for 24h and *CYP1A1* **(Left)** and *TIPARP* **(middle)** mRNA was quantified using RT-PCR. CT = control. **Lower Right:** HT-29 cells were pre-treated with CH-223191 for 3h and thereafter treated with 50µM BOLD-100 for 24h and ROS levels assessed using the ROS-Glo™ H_2O_2 Assay Kit. **E.** HT-29, VACO432 and VT1 cells were treated with with the indicated doses of Tapinarof for 24h. *CYP1A1* mRNA was quantified using RT-PCR. Raw values were normalised to the expression of housekeeping genes *ACTB* and *GAPDH* and were analysed using the ΔΔCT method. mRNA levels presented are relative to untreated control. CONT = control. **F.** Membrane (MF), cytosolic (CF) and nuclear (NF) fractions were isolated from CRC cells, and equal amounts of protein were immunoblotted for AhR, DR5, pMEK1/2$^{S217/221}$, MEK1/2 and PARP.
