# Supplementary material for: Ruthenium Drug BOLD-100 Regulates BRAFMT Colorectal Cancer Cell Apoptosis through AhR/ROS/ATR Signaling Axis Modulation
Source: Mol Cancer Res. 2024 Jul 31;22(12):1088–101. doi: 10.1158/1541-7786.MCR-24-0151 (PMC7616621; doi:10.1158/1541-7786.MCR-24-0151)
Supplement: Supplementary Mat & Methods — Supplementary M & M [file mcr-24-0151_supplementary_mat__methods_suppsm1.docx]

**The anticancer Ruthenium drug BOLD-100 regulates apoptosis of *BRAF*MT colorectal cancer cells through modulation of the AhR/ROS/ATR signalling axis.**

Daryl Griffin^1^, Robbie Carson^1^, Debbie Moss^1^, Tamas Sessler^1^, Deborah Lavin^1^, Vijay K. Tiwari^1,2^, Shivaali Karelia^1^, Richard Kennedy^1^, Kienan I. Savage^1^, Simon McDade^1^, Adam Carie^3^, Jim Pankovich^3^, Mark Bazett^3^, Sandra Van Schaeybroeck^1,4^

**SUPPLEMENTARY MATERIALS AND METHODS**

**FDA and Non-FDA approved compounds for drug screen**

FDA approved drugs for drug screen include Selumetinib (AZD6244) (1), Vemurafenib (2), Ruxolitinib (3), Entinostat (MS-275) (4), Vorinostat (SAHA, MK0683) (5), Cetuximab (6), Paclitaxel (7), Doxorubicin (8), Vincristine (9), Hydroxyurea (10), Trifluridine/tipiracil (TAS-102) (11), Palbociclib (12), Chloroambucil (13), Vinorelbine Tartrate (14), Decitabine (15), Lapatinib (16), Vismodegib (GDC-0449) (17), Everolimus (18) and Regorafenib (19) .

Non-FDA approved compounds used in this manuscript include ABT-737 (20), AZD1480 (21), AZD5363 (22), ACY*-*1215 (ricolinostat) (23), AZD5991 (24), GSK2606414 (25) , AZD0424 (26), KU60019 (27), recombinant human (rh)TRAIL (28), KU55933 (29), AZD7762 (30), AZD6738 (31), Mirin (32), BML-277 (33), NU7441 (34), Y-27632 2HCl (35), BI-2536 (36), Alisertib (MLN8237) (37), Roscovitine (38), Adavosertib (39), BS-181 (40), PHA-767491 (41), CVT-313 (42), SKPin C1 (43), Lomeguatrib (44), Dinaciclib (45), Nutlin-3 (46), Pinometostat (47), CPI-455 HCl (48), A-485 (49), Linsitinib (OSI-906) (50), YM-155 (51), Ganetespib (STA-9090) (52), Selisistat (EX-527) (53), Ixazomib (MLN2238) (54), Galunisertib (LY2157299) (55), Altiratinib (56), Wnt-C59 (57), RO-4929097 (58), SBI-0206965 (59), JNK-IN-8 (60), CHIR-99021 (61).

**Cell culture**

CX1, DLD-1, GP5D, HT-29, LOVO, LS174T, OXCO3, OUMS23, RW7213 and SW620 were cultured in DMEM, COLO205, HCC2998, HDC8, KM12, LIM1215, LIM2099, LIM2405, SNU-1411, LS513 and WiDR in RPMI-1640, HCT116, RKO, VACO432 in McCoys 5A, C125PM and C106 in IMDM and CCD18 in EMEM. Each media was supplemented with 10% FCS and 1mM sodium pyruvate.

**DNA expression constructs**

The *BRAF*V600E plasmid was a gift from Prof. Marais (London, UK)(62).

**Probes**

TaqMan probes were purchased from Thermo Fisher Scientific.

RT-PCR

CYP1A1: Hs01054796_g1

TIPARP: Hs00296054_m1

CYP1B1: Hs00164383_m1

LINC00511: Hs03659217_m1

AHR: Hs00169233_m1

RRM2: Hs00357247_g1

MCM7: Hs00428518_m1

FANC1: Hs00383049_m1

EXO1: Hs01116190_m1

FEN1: Hs00748727_s1

ACTB: Hs03023943_g1

GAPDH: Hs02786624_g1

**RNA-sequencing and analysis (GSE252858)**

Bulk RNA-sequencing was performed for VACO432 and VT1 cells treated with IC_30_ dose (24μM) BOLD-100 for 3h and 24h. Three biological replicates for each sample were included in the experiment. Total RNA was extracted as described in the main materials and methods section. Each sample underwent QC, and library preparation was performed with 100ng RNA (Illumina NextSeq 500 kit, Illumina, UK), according to the manufacturer's instructions. Using the KAPA RNA HyperPrep Kit with RiboErase (HMR) (Roche, USA), paired 91 end sequencing was conducted with a sequencing depth of 50 million reads, resulting in 75-base pair reads. The reads were trimmed based on Phred score from the 3-prime end and then aligned to the human genome (hg38) using TopHat21 (version 2.1.0) with default settings. Following library size normalization using DESeq2 (version 3.5), expression was quantified, and differential expression analysis was performed using the DESeq2 package (63). PCA plots, showing clusters of samples based on their similarity were checked to ensure differences in experimental groups. A cut-off threshold of fold-change >1.5 or <-1.5, and adj.p-value<0.05 was applied to generate gene lists (Supplementary Table S4). For volcano plots, log_2_ratios of time-point vs. control and -log_10_ adj.p-values for genes were plotted on the x- and y-axis respectively, in Prism 10.1. RNA-sequencing associated files have been deposited to the gene expression omnibus and are available to download under accession number GSE252858.

**siRNA screening**

A customised siRNA library targeting 177 tumour suppressor genes or genes whose loss of function was associated with cancer (Supplementary Table S2) was obtained using predesigned siRNA sequences (Qiagen). The initial library contained 1 siRNA sequence per gene, was provided in a 96-well arrayed format and transferred into a 384-well array format using the Echo525 (Beckman Coulter, UK). HCT116 CRC cells were reverse transfected with the siRNA library (Qiagen) using HiPerfect as per manufacturer’s instructions (Qiagen). Cells were transfected with 10nM siRNA. Twenty-four hours later, cells were treated with either DMSO or 50μM BOLD-100, and after 48h, cell viability was assayed using the Cell Titre Glo assay. The sensitivity of the screen was monitored by PLK1 siRNA causing a reduction in viability of more than 90%, when compared to viability of the non-targeting siRNA control (AllStars Negative Control). The siRNA screen was performed twice and mean robust-Z scores were calculated per gene. Genes with robust Z-scores of >1 or <-1, were carried forward in a secondary screen, containing 2 additional siRNA sequences per gene (Supplementary Table S2). The siRNA secondary screen was performed twice and mean robust Z-scores of the 2 independent siRNAs targeting each gene was also determined. Genes with mean robust Z-scores >1 or <-1 for each of the 3 independent siRNA’s (primary and secondary screen), were carried forward for further validation.

***In vivo* study**

Tolerability study

The maximum tolerated dose of BOLD-100 (intravenous; 50mg/kg) in combination with AZD6738 (oral gavage; 12.5, 25 or 50mg/kg), was assessed in Balb/c nude mice in a dose-escalation study. BOLD-100 was formulated in 0.9% saline buffer with 10 mM citrate buffer, AZD6738 in 10% DMSO + 90% HPβCD (10% made in PBS). BOLD-100 was administered weekly by intravenous injection on treatment days 1 and 8, and AZD6738 was administered once daily by oral gavage on treatment days 1 to 14. The concentration of BOLD-100 used in this study was 50mg/kg. The concentration of AZD6738 used in this study started at 12.5 mg/kg and increased to 25mg/kg and 50mg/kg when tolerability was observed. Outward signs of distress and mouse weight were monitored daily was monitored. MTD was defined as the maximal dose of both drugs in combination which does not results in weight loss > 15% or death (Supplementary Fig. S4D).

**REFERENCES**

1. Garon EB, Finn RS, Hosmer W, Dering J, Ginther C, Adhami S, et al. Identification of common predictive markers of in vitro response to the Mek inhibitor selumetinib (AZD6244; ARRY-142886) in human breast cancer and non-small cell lung cancer cell lines. Mol Cancer Ther 2010; 9: 1985-94.

2. Halaban R, Zhang W, Bacchiocchi A, Cheng E, Parisi F, Ariyan S, et al. PLX4032, a selective BRAF(V600E) kinase inhibitor, activates the ERK pathway and enhances cell migration and proliferation of BRAF melanoma cells. Pigment Cell Melanoma Res 2010; 23: 190-200.

3. Mesa RA. Ruxolitinib, a selective JAK1 and JAK2 inhibitor for the treatment of myeloproliferative neoplasms and psoriasis. IDrugs 2010; 13: 394-403.

4. Rao-Bindal K, Koshkina NV, Stewart J,Kleinerman ES. The histone deacetylase inhibitor, MS-275 (entinostat), downregulates c-FLIP, sensitizes osteosarcoma cells to FasL, and induces the regression of osteosarcoma lung metastases. Curr Cancer Drug Targets 2013; 13: 411-22.

5. Finnin MS, Donigian JR, Cohen A, Richon VM, Rifkind RA, Marks PA, et al. Structures of a histone deacetylase homologue bound to the TSA and SAHA inhibitors. Nature 1999; 401: 188-93.

6. Jonker DJ, O'Callaghan CJ, Karapetis CS, Zalcberg JR, Tu D, Au HJ, et al. Cetuximab for the treatment of colorectal cancer. N Engl J Med 2007; 357: 2040-8.

7. Takayama K, Ichiki M, Tokunaga S, Inoue K, Kawasaki M, Uchino J, et al. Randomized Phase II Study of Weekly Paclitaxel plus Carboplatin Versus Biweekly Paclitaxel plus Carboplatin for Patients with Previously Untreated Advanced Non-Small Cell Lung Cancer. Oncologist 2019; 24: 1420-e010.

8. Judson I, Verweij J, Gelderblom H, Hartmann JT, Schoffski P, Blay JY, et al. Doxorubicin alone versus intensified doxorubicin plus ifosfamide for first-line treatment of advanced or metastatic soft-tissue sarcoma: a randomised controlled phase 3 trial. Lancet Oncol 2014; 15: 415-23.

9. Feng F, Xiang Y, Wan X, Geng S,Wang T. Salvage combination chemotherapy with floxuridine, dactinomycin, etoposide, and vincristine (FAEV) for patients with relapsed/chemoresistant gestational trophoblastic neoplasia. Ann Oncol 2011; 22: 1588-94.

10. Charache S, Terrin ML, Moore RD, Dover GJ, McMahon RP, Barton FB, et al. Design of the multicenter study of hydroxyurea in sickle cell anemia. Investigators of the Multicenter Study of Hydroxyurea. Control Clin Trials 1995; 16: 432-46.

11. Mayer RJ, Van Cutsem E, Falcone A, Yoshino T, Garcia-Carbonero R, Mizunuma N, et al. Randomized trial of TAS-102 for refractory metastatic colorectal cancer. N Engl J Med 2015; 372: 1909-19.

12. Turkington RC, Longley DB, Allen WL, Stevenson L, McLaughlin K, Dunne PD, et al. Fibroblast growth factor receptor 4 (FGFR4): a targetable regulator of drug resistance in colorectal cancer. Cell death & disease 2014; 5: e1046.

13. Goede V, Fischer K, Busch R, Engelke A, Eichhorst B, Wendtner CM, et al. Obinutuzumab plus chlorambucil in patients with CLL and coexisting conditions. N Engl J Med 2014; 370: 1101-10.

14. Toso C,Lindley C. Vinorelbine: a novel vinca alkaloid. Am J Health Syst Pharm 1995; 52: 1287-304; quizz 340-1.

15. Welch JS, Petti AA, Miller CA, Fronick CC, O'Laughlin M, Fulton RS, et al. TP53 and Decitabine in Acute Myeloid Leukemia and Myelodysplastic Syndromes. N Engl J Med 2016; 375: 2023-36.

16. Geyer CE, Forster J, Lindquist D, Chan S, Romieu CG, Pienkowski T, et al. Lapatinib plus capecitabine for HER2-positive advanced breast cancer. N Engl J Med 2006; 355: 2733-43.

17. Sekulic A, Migden MR, Oro AE, Dirix L, Lewis KD, Hainsworth JD, et al. Efficacy and safety of vismodegib in advanced basal-cell carcinoma. N Engl J Med 2012; 366: 2171-9.

18. Yao JC, Shah MH, Ito T, Bohas CL, Wolin EM, Van Cutsem E, et al. Everolimus for advanced pancreatic neuroendocrine tumors. N Engl J Med 2011; 364: 514-23.

19. Grothey A, Van Cutsem E, Sobrero A, Siena S, Falcone A, Ychou M, et al. Regorafenib monotherapy for previously treated metastatic colorectal cancer (CORRECT): an international, multicentre, randomised, placebo-controlled, phase 3 trial. Lancet 2013; 381: 303-12.

20. Kline MP, Rajkumar SV, Timm MM, Kimlinger TK, Haug JL, Lust JA, et al. ABT-737, an inhibitor of Bcl-2 family proteins, is a potent inducer of apoptosis in multiple myeloma cells. Leukemia 2007; 21: 1549-60.

21. Hedvat M, Huszar D, Herrmann A, Gozgit JM, Schroeder A, Sheehy A, et al. The JAK2 inhibitor AZD1480 potently blocks Stat3 signaling and oncogenesis in solid tumors. Cancer Cell 2009; 16: 487-97.

22. Davies BR, Greenwood H, Dudley P, Crafter C, Yu DH, Zhang J, et al. Preclinical pharmacology of AZD5363, an inhibitor of AKT: pharmacodynamics, antitumor activity, and correlation of monotherapy activity with genetic background. Mol Cancer Ther 2012; 11: 873-87.

23. Santo L, Hideshima T, Kung AL, Tseng JC, Tamang D, Yang M, et al. Preclinical activity, pharmacodynamic, and pharmacokinetic properties of a selective HDAC6 inhibitor, ACY-1215, in combination with bortezomib in multiple myeloma. Blood 2012; 119: 2579-89.

24. Tron AE, Belmonte MA, Adam A, Aquila BM, Boise LH, Chiarparin E, et al. Discovery of Mcl-1-specific inhibitor AZD5991 and preclinical activity in multiple myeloma and acute myeloid leukemia. Nature communications 2018; 9: 5341.

25. Axten JM, Romeril SP, Shu A, Ralph J, Medina JR, Feng Y, et al. Discovery of GSK2656157: An Optimized PERK Inhibitor Selected for Preclinical Development. ACS medicinal chemistry letters 2013; 4: 964-8.

26. Dawson JC, Munro A, Macleod K, Muir M, Timpson P, Williams RJ, et al. Pathway profiling of a novel SRC inhibitor, AZD0424, in combination with MEK inhibitors for cancer treatment. Molecular oncology 2022; 16: 1072-90.

27. Golding SE, Rosenberg E, Valerie N, Hussaini I, Frigerio M, Cockcroft XF, et al. Improved ATM kinase inhibitor KU-60019 radiosensitizes glioma cells, compromises insulin, AKT and ERK prosurvival signaling, and inhibits migration and invasion. Mol Cancer Ther 2009; 8: 2894-902.

28. Khawaja H, Campbell A, Roberts JZ, Javadi A, O'Reilly P, McArt D, et al. RALB GTPase: a critical regulator of DR5 expression and TRAIL sensitivity in KRAS mutant colorectal cancer. Cell death & disease 2020; 11: 930.

29. Hickson I, Zhao Y, Richardson CJ, Green SJ, Martin NM, Orr AI, et al. Identification and characterization of a novel and specific inhibitor of the ataxia-telangiectasia mutated kinase ATM. Cancer Res 2004; 64: 9152-9.

30. Mitchell JB, Choudhuri R, Fabre K, Sowers AL, Citrin D, Zabludoff SD, et al. In vitro and in vivo radiation sensitization of human tumor cells by a novel checkpoint kinase inhibitor, AZD7762. Clin Cancer Res 2010; 16: 2076-84.

31. Vendetti FP, Lau A, Schamus S, Conrads TP, O'Connor MJ,Bakkenist CJ. The orally active and bioavailable ATR kinase inhibitor AZD6738 potentiates the anti-tumor effects of cisplatin to resolve ATM-deficient non-small cell lung cancer in vivo. Oncotarget 2015; 6: 44289-305.

32. Dupre A, Boyer-Chatenet L, Sattler RM, Modi AP, Lee JH, Nicolette ML, et al. A forward chemical genetic screen reveals an inhibitor of the Mre11-Rad50-Nbs1 complex. Nat Chem Biol 2008; 4: 119-25.

33. Hsieh CC, Hsu SH, Lin CY, Liaw HJ, Li TW, Jiang KY, et al. CHK2 activation contributes to the development of oxaliplatin resistance in colorectal cancer. Br J Cancer 2022; 127: 1615-28.

34. Zhao Y, Thomas HD, Batey MA, Cowell IG, Richardson CJ, Griffin RJ, et al. Preclinical evaluation of a potent novel DNA-dependent protein kinase inhibitor NU7441. Cancer Res 2006; 66: 5354-62.

35. Sivasubramaniyan K, Pal R, Totey S, Bhat VS,Totey S. Rho kinase inhibitor y27632 alters the balance between pluripotency and early differentiation events in human embryonic stem cells. Curr Stem Cell Res Ther 2010; 5: 2-12.

36. Steegmaier M, Hoffmann M, Baum A, Lenart P, Petronczki M, Krssak M, et al. BI 2536, a potent and selective inhibitor of polo-like kinase 1, inhibits tumor growth in vivo. Current biology : CB 2007; 17: 316-22.

37. Manfredi MG, Ecsedy JA, Chakravarty A, Silverman L, Zhang M, Hoar KM, et al. Characterization of Alisertib (MLN8237), an investigational small-molecule inhibitor of aurora A kinase using novel in vivo pharmacodynamic assays. Clin Cancer Res 2011; 17: 7614-24.

38. Havlicek L, Hanus J, Vesely J, Leclerc S, Meijer L, Shaw G, et al. Cytokinin-derived cyclin-dependent kinase inhibitors: synthesis and cdc2 inhibitory activity of olomoucine and related compounds. Journal of medicinal chemistry 1997; 40: 408-12.

39. Liu JF, Xiong N, Campos SM, Wright AA, Krasner C, Schumer S, et al. Phase II Study of the WEE1 Inhibitor Adavosertib in Recurrent Uterine Serous Carcinoma. J Clin Oncol 2021; 39: 1531-9.

40. Wang BY, Liu QY, Cao J, Chen JW,Liu ZS. Selective CDK7 inhibition with BS-181 suppresses cell proliferation and induces cell cycle arrest and apoptosis in gastric cancer. Drug Des Devel Ther 2016; 10: 1181-9.

41. Li W, Zhao XL, Shang SQ, Shen HQ,Chen X. Dual Inhibition of Cdc7 and Cdk9 by PHA-767491 Suppresses Hepatocarcinoma Synergistically with 5-Fluorouracil. Curr Cancer Drug Targets 2015; 15: 196-204.

42. Brooks EE, Gray NS, Joly A, Kerwar SS, Lum R, Mackman RL, et al. CVT-313, a specific and potent inhibitor of CDK2 that prevents neointimal proliferation. J Biol Chem 1997; 272: 29207-11.

43. Yang Y, Yan W, Liu Z,Wei M. Skp2 inhibitor SKPin C1 decreased viability and proliferation of multiple myeloma cells and induced apoptosis. Braz J Med Biol Res 2019; 52: e8412.

44. Ranson M, Middleton MR, Bridgewater J, Lee SM, Dawson M, Jowle D, et al. Lomeguatrib, a potent inhibitor of O6-alkylguanine-DNA-alkyltransferase: phase I safety, pharmacodynamic, and pharmacokinetic trial and evaluation in combination with temozolomide in patients with advanced solid tumors. Clin Cancer Res 2006; 12: 1577-84.

45. Pomeroy EJ, Lee LA, Lee RDW, Schirm DK, Temiz NA, Ma J, et al. Ras oncogene-independent activation of RALB signaling is a targetable mechanism of escape from NRAS(V12) oncogene addiction in acute myeloid leukemia. Oncogene 2017; 36: 3263-73.

46. Vassilev LT, Vu BT, Graves B, Carvajal D, Podlaski F, Filipovic Z, et al. In vivo activation of the p53 pathway by small-molecule antagonists of MDM2. Science 2004; 303: 844-8.

47. Campbell CT, Haladyna JN, Drubin DA, Thomson TM, Maria MJ, Yamauchi T, et al. Mechanisms of Pinometostat (EPZ-5676) Treatment-Emergent Resistance in MLL-Rearranged Leukemia. Mol Cancer Ther 2017; 16: 1669-79.

48. Vinogradova M, Gehling VS, Gustafson A, Arora S, Tindell CA, Wilson C, et al. An inhibitor of KDM5 demethylases reduces survival of drug-tolerant cancer cells. Nat Chem Biol 2016; 12: 531-8.

49. Ji C, Xu W, Ding H, Chen Z, Shi C, Han J, et al. The p300 Inhibitor A-485 Exerts Antitumor Activity in Growth Hormone Pituitary Adenoma. The Journal of clinical endocrinology and metabolism 2022; 107: e2291-e300.

50. Mulvihill MJ, Cooke A, Rosenfeld-Franklin M, Buck E, Foreman K, Landfair D, et al. Discovery of OSI-906: a selective and orally efficacious dual inhibitor of the IGF-1 receptor and insulin receptor. Future Med Chem 2009; 1: 1153-71.

51. Voges Y, Michaelis M, Rothweiler F, Schaller T, Schneider C, Politt K, et al. Effects of YM155 on survivin levels and viability in neuroblastoma cells with acquired drug resistance. Cell death & disease 2016; 7: e2410.

52. Goldman JW, Raju RN, Gordon GA, El-Hariry I, Teofilivici F, Vukovic VM, et al. A first in human, safety, pharmacokinetics, and clinical activity phase I study of once weekly administration of the Hsp90 inhibitor ganetespib (STA-9090) in patients with solid malignancies. BMC Cancer 2013; 13: 152.

53. Gertz M, Fischer F, Nguyen GT, Lakshminarasimhan M, Schutkowski M, Weyand M, et al. Ex-527 inhibits Sirtuins by exploiting their unique NAD+-dependent deacetylation mechanism. Proc Natl Acad Sci U S A 2013; 110: E2772-81.

54. Shirley M. Ixazomib: First Global Approval. Drugs 2016; 76: 405-11.

55. Dituri F, Mazzocca A, Fernando J, Peidro FJ, Papappicco P, Fabregat I, et al. Differential Inhibition of the TGF-beta Signaling Pathway in HCC Cells Using the Small Molecule Inhibitor LY2157299 and the D10 Monoclonal Antibody against TGF-beta Receptor Type II. PLoS One 2013; 8: e67109.

56. Smith BD, Kaufman MD, Leary CB, Turner BA, Wise SC, Ahn YM, et al. Altiratinib Inhibits Tumor Growth, Invasion, Angiogenesis, and Microenvironment-Mediated Drug Resistance via Balanced Inhibition of MET, TIE2, and VEGFR2. Mol Cancer Ther 2015; 14: 2023-34.

57. Motono M, Ioroi Y, Ogura T,Takahashi J. WNT-C59, a Small-Molecule WNT Inhibitor, Efficiently Induces Anterior Cortex That Includes Cortical Motor Neurons From Human Pluripotent Stem Cells. Stem Cells Transl Med 2016; 5: 552-60.

58. Luistro L, He W, Smith M, Packman K, Vilenchik M, Carvajal D, et al. Preclinical profile of a potent gamma-secretase inhibitor targeting notch signaling with in vivo efficacy and pharmacodynamic properties. Cancer Res 2009; 69: 7672-80.

59. Dower CM, Bhat N, Gebru MT, Chen L, Wills CA, Miller BA, et al. Targeted Inhibition of ULK1 Promotes Apoptosis and Suppresses Tumor Growth and Metastasis in Neuroblastoma. Mol Cancer Ther 2018; 17: 2365-76.

60. Zhang T, Inesta-Vaquera F, Niepel M, Zhang J, Ficarro SB, Machleidt T, et al. Discovery of potent and selective covalent inhibitors of JNK. Chemistry & biology 2012; 19: 140-54.

61. Liang W, Cho HC,Marban E. Wnt signalling suppresses voltage-dependent Na(+) channel expression in postnatal rat cardiomyocytes. J Physiol 2015; 593: 1147-57.

62. Marais R, Light Y, Paterson HF, Mason CS,Marshall CJ. Differential regulation of Raf-1, A-Raf, and B-Raf by oncogenic ras and tyrosine kinases. J Biol Chem 1997; 272: 4378-83.

63. Love MI, Huber W,Anders S. Moderated estimation of fold change and dispersion for RNA-seq data with DESeq2. Genome Biol 2014; 15: 550.
